# Tensor Analysis Reveals Distinct Population Structure that Parallels the Different Computational Roles of Areas M1 and V1

**DOI:** 10.1371/journal.pcbi.1005164

**Published:** 2016-11-04

**Authors:** Jeffrey S. Seely, Matthew T. Kaufman, Stephen I. Ryu, Krishna V. Shenoy, John P. Cunningham, Mark M. Churchland

**Affiliations:** 1 Department of Neuroscience, Columbia University Medical Center, New York, NY, United States of America; 2 Neurosciences Program,Stanford University, Stanford, CA, United States of America; 3 Department of Electrical Engineering, Stanford University, Stanford, CA, United States of America; 4 Cold Spring Harbor Laboratory, Cold Spring Harbor, NY, United States of America; 5 Department of Neurosurgery, Palo Alto Medical Foundation, Palo Alto, CA, United States of America; 6 Department of Bioengineering, Stanford University, Stanford, CA, United States of America; 7 Department of Neurobiology, Stanford University, Stanford, CA, United States of America; 8 Stanford Neurosciences Institute, Stanford University, Stanford, CA, United States of America; 9 Howard Hughes Medical Institute Stanford University, Stanford, CA, United States of America; 10 Grossman Center for the Statistics of Mind, Columbia University Medical Center, New York, NY, United States of America; 11 Department of Statistics, Columbia University, New York, NY, United States of America; 12 David Mahoney Center for Brain and Behavior Research, Columbia University Medical Center, New York, NY, United States of America; 13 Kavli Institute for Brain Science, Columbia University Medical Center, New York, NY, United States of America; University College London, UNITED KINGDOM

## Abstract

Cortical firing rates frequently display elaborate and heterogeneous temporal structure. One often wishes to compute quantitative summaries of such structure—a basic example is the frequency spectrum—and compare with model-based predictions. The advent of large-scale population recordings affords the opportunity to do so in new ways, with the hope of distinguishing between potential explanations for why responses vary with time. We introduce a method that assesses a basic but previously unexplored form of population-level structure: when data contain responses across multiple neurons, conditions, and times, they are naturally expressed as a third-order tensor. We examined tensor structure for multiple datasets from primary visual cortex (V1) and primary motor cortex (M1). All V1 datasets were ‘simplest’ (there were relatively few degrees of freedom) along the neuron mode, while all M1 datasets were simplest along the condition mode. These differences could not be inferred from surface-level response features. Formal considerations suggest why tensor structure might differ across modes. For idealized linear models, structure is simplest across the neuron mode when responses reflect external variables, and simplest across the condition mode when responses reflect population dynamics. This same pattern was present for existing models that seek to explain motor cortex responses. Critically, only dynamical models displayed tensor structure that agreed with the empirical M1 data. These results illustrate that tensor structure is a basic feature of the data. For M1 the tensor structure was compatible with only a subset of existing models.

## Author Summary

Neuroscientists commonly measure the time-varying activity of neurons in the brain. Early studies explored how such activity directly encodes sensory stimuli. Since then neural responses have also been found to encode abstract parameters such as expected reward. Yet not all aspects of neural activity directly encode identifiable parameters: patterns of activity sometimes reflect the evolution of underlying internal computations, and may be only obliquely related to specific parameters. For example, it remains debated whether cortical activity during movement relates to parameters such as reach velocity, to parameters such as muscle activity, or to underlying computations that culminate in the production of muscle activity. To address this question we exploited an unexpected fact. When activity directly encodes a parameter it tends to be mathematically simple in a very particular way. When activity reflects the evolution of a computation being performed by the network, it tends to be mathematically simple in a different way. We found that responses in a visual area were simple in the first way, consistent with encoding of parameters. We found that responses in a motor area were simple in the second way, consistent with participation in the underlying computations that culminate in movement.

## Introduction

Cortical neurons often display temporally complex firing rate patterns (*e*.*g*., [1,2]). Such temporal structure may have at least two non-exclusive sources. First, temporal structure may reflect external variables that drive or are being encoded by the population; *e*.*g*., a time-varying stimulus or a time-varying parameter represented by the population [3,4]. Second, temporal structure may reflect internal population-level dynamics. For example, oscillatory responses are observed in isolated spinal populations [5], and even sensory areas exhibit response transients due to cellular and network dynamics [6]. One often wishes to disentangle the contributions of external variables and internal dynamics. Yet without full knowledge of the relevant external variables, response patterns can in principle originate from either source [7]. For example, a sinusoidal response might reflect a sinusoidal external variable, oscillatory population dynamics, or both.

Motor cortex (M1) presents a paradigmatic example where temporal response complexity [1,8–10] has fed a long-standing debate [11–21]. Guided by one viewpoint, many studies have focused on the possibility that M1 responses reflect specific external behavioral variables, and have sought to determine their identity (reach direction, velocity, joint torques, muscle forces, etc. [21]) and reference frame [22–28]. Guided by another viewpoint, recent studies suggest that the temporal structure of M1 responses may largely reflect the evolution of internal population dynamics [29–33]. This second viewpoint is embodied in recurrent network models of pattern generation [34–36], and is broadly compatible with control-theory models [37–39] where dynamics may involve both internal recurrence and feedback.

While not necessarily opposed, the first and second viewpoints often make different predictions even when starting with shared assumptions. Suppose one began with the assumption that, during reaching, motor cortex controls muscle activity more-or-less directly [14]. The first viewpoint predicts that neural responses will be a function of (will ‘encode’) the patterns of muscle activity. The first viewpoint does not predict that neural responses should obey dynamics: the future neural state would not be a consistent function of the present neural state. While muscle activity is ‘dynamic’ in the sense that it is time-varying, it is not typically true that the set of muscle activations obeys a single dynamical system (*i*.*e*. a fixed flow field) across different reaches. The second viewpoint, in contrast, predicts that the motor cortex population response should obey consistent dynamics. The second viewpoint, like the first, predicts that muscle activity will be a function of neural responses [40,41]. Yet because that function is presumably non-invertible, neural responses will not be a function of muscle activity, in opposition to the first viewpoint.

The hypothesis that neural responses reflect external variables (*e*.*g*., muscle activity itself) and the hypothesis that neural responses reflect internal dynamics (*e*.*g*., the dynamics that produce muscle activity) could be readily distinguished were it known that muscle activity was the relevant external variable. However, that assumption is itself the subject of controversy [8,14,15,17,27,40,42–45]. It therefore remains debated whether M1 response structure originates from a representation of external movement variables or the unfolding of internal dynamics. Recent experimental studies [30,46] and reviews [19,32] have advanced both positions.

Motor cortex thus illustrates a general need: the ability to infer the predominant origin of time-varying responses. We report here that a basic but previously unmeasured feature of neural population data is surprisingly informative to this need. We considered the population response as a third-order tensor (a three-dimensional array) indexed by neuron, condition and time. We were motivated by the idea that tuning for external variables constrains structure across neurons; if there are ten relevant external variables, responses are limited to ten degrees of freedom across neurons. We refer to this setting as ‘neuron-preferred.’ Conversely, internal dynamics constrain structure across conditions; if a population obeys the same dynamics across conditions, responses will have limited degrees of freedom across conditions. We refer to this situation as ‘condition-preferred.’ Neuron-preferred or condition-preferred structure is hidden at both the single-neuron level and in standard population-level analyses—*i*.*e*. this structure is hidden if the data is viewed only as a matrix.

Intuitions regarding neuron-preferred versus condition-preferred structure can be gained by considering linear models. For example, the input-driven system

x(c,t)=Bu(c,t), (1)

and the autonomous dynamical system

x(c,t+1)=Ax(c,t), (2)

can be viewed as two different generators of a data tensor $X\in {\mathbb{R}}^{N\times C\times T}$, with *x*(*c*,*t*) ∈ ℝ^*N*^ the vector of *N* neural responses at time *t* for condition *c*, *u*(*c*,*t*) ∈ ℝ^*M*^ the vector of *M* input variables, *B* ∈ ℝ^*N*×*M*^, and *A* ∈ ℝ^*N*×*N*^. Time-varying structure of X generated by the first equation is inherited from the time-varying structure of *u*(*c*,*t*), while for the second it is inherited from the time-varying structure of *A*^*t*^, since Eq (2) can be expressed as *x*(*c*,*t*) = *A*^*t*^*x*(*c*,0). As will be formalized later, neuron-preferred tensor structure follows naturally from Eq (1): each *C* × *T* ‘slice’ of the data tensor X (*i*.*e*., the data for a given neuron across all conditions and times) is a linear combination of a bounded number of basis elements, each of size *C* × *T*. Condition-preferred structure follows naturally from Eq (2): each *N* × *T* ‘slice’ of the data tensor X (*i*.*e*., the data for a given condition across all neurons and times) is a linear combination of a bounded number of basis elements, each of size *N* × *T*. We choose the term ‘neuron-preferred’ to describe the case where there are fewer degrees of freedom across neurons, and the term ‘condition-preferred’ to describe the case where there are fewer degrees of freedom across conditions. Thus, the ‘preferred mode’ is the mode (neuron or condition) from which the data tensor can be most accurately reconstructed using the smallest number of basis elements.

Our investigation of the preferred mode was guided by a three-part hypothesis. First, we hypothesized that empirical population responses may often have a clear preferred mode. Second, we hypothesized that the preferred mode likely differs between brain areas. To address these hypotheses, we assessed the preferred mode for three neural datasets recorded from primary visual cortex (V1) and four neural datasets recorded from M1. V1 datasets were strongly neuron-preferred, while M1 datasets were strongly condition-preferred. Third, we hypothesized that the preferred mode might be informative regarding the origin of population responses. We concentrated on models of M1, and found that existing models based on tuning for external variables were neuron-preferred, in opposition to the M1 data. However, existing models with strong internal dynamics were condition-preferred, in agreement with the data. Model success or failure depended not on parameter choice or fit quality, but on model class. We conclude that tensor structure is informative regarding the predominant origin of time-varying activity, and can be used to test specific hypotheses. In the present case, the tensor structure of M1 datasets is consistent with only a subset of existing models.

## Results

### Time-varying response structure

We analyzed nine physiological datasets: three recorded from V1 during presentation of visual stimuli, four recorded from M1 during reaching tasks, and two recorded from muscle populations during the same reaching tasks. Each dataset employed multiple conditions: different stimuli/reaches. Each neuron’s response was averaged across trials within a condition and smoothed to produce a firing rate as a function of time. Some recordings were simultaneous and some were sequential, but in all cases the same set of conditions was employed for every neuron. Stimuli were never tailored to individual neurons (*e*.*g*., to their preferred direction or receptive field). This allows for analysis of the true population response, indexed by neuron, condition, and time. For the muscle populations, electromyographic (EMG) voltages were converted to a smooth function of intensity versus time via standard rectification and filtering. Muscle populations were then analyzed in the same way as neural populations, but individual elements were muscles rather than neurons. We analyzed ten further datasets simulated using existing models of M1.

**Fig 1:**
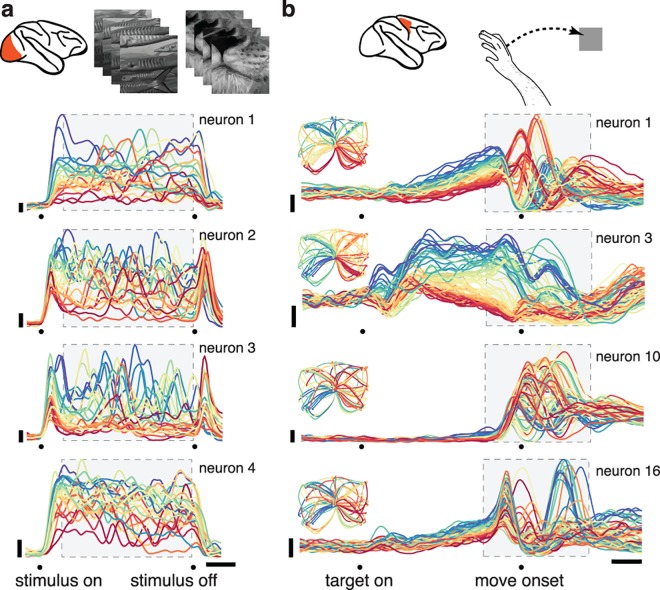
Illustration of the stimuli/task and neural responses for one V1 dataset and one M1 dataset. (**a**) Responses of four example neurons for a V1 dataset recorded via an implanted electrode array during presentation of movies of natural scenes. Each colored trace plots the trial-averaged firing rate for one condition (one of 25 movies). For visualization, traces are colored red to blue based on the firing rate early in the stimulus. (**b**) Responses of four example neurons for an M1 dataset recorded via two implanted electrode arrays during a delayed-reach task (monkey J). Example neurons were chosen to illustrate the variety of observed responses. Each colored trace plots the trial-averaged firing rate for one condition; *i*.*e*., one of 72 straight and curved reach trajectories. For visualization, traces are colored based on the firing rate during the delay period between target onset and the go cue. Insets show the reach trajectories (which are the same for each neuron) using the color-coding for that neuron. M1 responses were time-locked separately to the three key events: target onset, the go cue, and reach onset. For presentation, the resulting average traces were spliced together to create a continuous firing rate as a function of time. However, the analysis window included primarily movement-related activity. Gray boxes indicate the analysis windows (for V1, *T* = 91 time points spanning 910 ms; for M1, *T* = 71 time points spanning 710 ms). Horizontal bars: 200 ms; vertical bars: 20 spikes per second.

We first focus on two datasets: one from V1 (**Fig 1A**) and one from M1 (**Fig 1B**). The V1 dataset was recorded using a 96-electrode array from an anesthetized monkey viewing one-second movies of natural scenes (25 movies, 50 trials each). The M1 dataset was recorded using a pair of implanted 96-electrode arrays, spanning the arm representation of primary motor cortex and the immediately adjacent region of dorsal premotor cortex (all results were similar if primary motor and premotor cortex were treated separately). Neural responses were recorded during a delayed reach task: the monkey touched a central spot on a screen, was presented with a target, then executed a reach following a go cue. We analyzed data for 72 conditions (**Fig 1B**, insets), each involving a different reach distance and curvature (average of 28 trials per condition) [30].

Both V1 and M1 neurons displayed temporally complex response patterns (**Fig 1**). Each colored trace plots the trial-averaged firing rate over time for one condition: a particular movie (**Fig 1A**) or reach (**Fig 1B**). V1 neurons exhibited multiphasic responses throughout the stimulus. M1 neurons exhibited multiphasic activity over a ~700 ms period that began shortly after the go cue. Tight standard error bars (not displayed) confirmed that temporal response structure was statistically reliable rather than the result of sampling noise. In M1 it has been debated whether such structure primarily reflects external factors such as reach kinematics or primarily reflects internal dynamics. Both hypotheses can claim support from surface-level features of the data. Responses vary strongly with reach kinematics (insets show reach trajectories color-coded according to the response properties of the neuron in that panel) as proposed by the first hypothesis. On the other hand, responses show some quasi-oscillatory features that could reflect underlying dynamics. Might a comparison with V1—where responses are known to be largely externally driven—be illuminating regarding the source of temporal response structure in M1?

V1 and M1 responses differed in a number of straightforward ways including frequency content and the overall response envelope. Such differences are expected given the different pacing of the task and stimuli. We wondered whether V1 and M1 datasets might also differ in deeper ways that are hidden at the level of the single neuron but clear at the level of the population. In general, a population response can differ across neurons, conditions, and time. While structure across time can be partially appreciated via inspection of single neurons (as in **Fig 1**), the joint structure across neurons and conditions is less patent. Are some datasets more constrained across neurons (‘neuron preferred’) and others more constrained across conditions (‘condition preferred’)? If so, might that carry implications?

### Preferred-mode analysis of V1 and M1

Neural population data is often analyzed in matrix form, allowing a number of standard analyses. Such analyses include assessing covariance structure and applying principal component analysis to extract the most prevalent response patterns [47]. One can then quantify, for a given number of extracted response patterns, how well they reconstruct the original data. This can provide a rough estimate of the number of degrees of freedom in the data [48].

**Fig 2:**
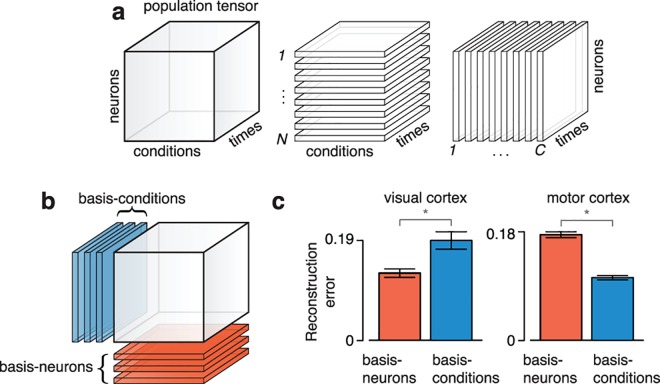
Schematic illustration of population tensor and results of a simplified preferred-mode analysis for two datasets. (**a**) The population response can be represented as firing rate values arranged in an *N* × *C* × *T* array, *i*.*e*. a third-order tensor indexed by neuron, condition, and time. That population tensor (left) can be thought of as a collection of *C* × *T* matrices (one for each neuron, middle) or a collection of *N* × *T* matrices (one for each condition, right). (**b**) The population tensor may be approximately reconstructed (via linear combinations) from a set of ‘basis-neurons’ (*C* × *T* matrices, red) or from a set of ‘basis-conditions’ (*N* × *T* matrices, blue). Depending on the nature of the data, the basis-neurons or the basis-conditions may provide the better reconstruction. (**c**) Normalized reconstruction error of the population tensors for the V1 and M1 datasets shown in **Fig 1**when reconstructed using basis neurons (red) or basis conditions (blue). Error bars show the standard errors across conditions (Methods). The number of basis elements (12 for V1 and 25 for M1) was the same for the neuron and condition modes and was chosen algorithmically (Methods). Robustness of the preferred mode with respect to the number of basis elements is shown in subsequent analyses.

However, when recordings span multiple neurons, conditions and times, the data are naturally formulated not as a matrix but as a third-order tensor of size *N* × *C* × *T*, where *N* is the number of neurons, *C* is the number of conditions, and *T* is the number of times. Each of these three indices is referred to as a ‘mode’ [49]. One can consider an *N* × *C* × *T* tensor as a collection of *N* matrices, each of size *C* × *T* (one per neuron), or as a collection of *C* matrices, each of size *N* × *T* (one per condition) (**Fig 2A**). One can then reconstruct the population tensor in two ways. First, one can reconstruct the responses of each neuron as a linear combination of a small collection of ‘basis-neurons,’ each of size *C* × *T* (**Fig 2B**, red matrices). Second, one can reconstruct each condition as a linear combination of a small collection of ‘basis-conditions,’ each of size *N* × *T* (**Fig 2B**, blue matrices). Unlike in the matrix case, for tensors a ‘preferred mode’ can exist.

To assess the preferred mode we applied the singular value decomposition (SVD) to the neuron and condition modes of the population tensor (Methods), yielding a set of basis-neurons and a set of basis-conditions. Performing SVD along a mode of a tensor, $X\in {\mathbb{R}}^{N\times C\times T}$, equates to performing SVD on one of the tensor’s matrix ‘unfoldings.’ We define the ‘mode-1’ and ‘mode-2’ unfolding of X as

$$X_{\left(1\right)}≔[\begin{array}{cccc} X(1) & X(2) & \cdots & X(T) \end{array}]\in {\mathbb{R}}^{N\times CT},$$ (3)

$$X_{\left(2\right)}≔[\begin{array}{cccc} X{\left(1\right)}^{⊤} & X{\left(2\right)}^{⊤} & \cdots & X{\left(T\right)}^{⊤} \end{array}]\in {\mathbb{R}}^{C\times NT},$$

where *X*(*t*) ∈ ℝ^*N*×*C*^ is the *N* × *C* matrix slice of X at time *t*. Each row of ***X***_(1)_ corresponds to one neuron, and each row of ***X***_(2)_ corresponds to one condition. The top *k* right singular vectors of ***X***_(1)_ are of dimension *CT*, thus can be reshaped to *C* × *T* matrices, corresponding to *k* basis-neurons. Similarly, the top *k* right singular vectors of ***X***_(2)_ are of dimension *NT* and can be reshaped to *N* × *T* matrices, corresponding to *k* basis-conditions. In this way each neuron (*i*.*e*., each row of ***X***_(1)_ and the corresponding *C* × *T* slice of X) can be approximately reconstructed as a linear combination of *k* basis-neurons. Similarly, each condition (*i*.*e*., each row of ***X***_(2)_ and the corresponding *N* × *T* slice of X) can be approximately reconstructed as a linear combination of *k* basis-conditions.

To assess the preferred mode we reconstructed each population tensor twice: once using a fixed number (*k*) of basis-neurons, and once using the same fixed number (*k*) of basis-conditions. Reconstruction error was the normalized squared error between the reconstructed tensor and the original data tensor. If basis-neurons provided the better reconstruction, the neuron mode was considered preferred. If basis-conditions provided the better reconstruction, the condition mode was considered preferred. (We explain later the algorithm for choosing the number of basis elements *k*, and explore robustness with respect to that choice).

The above procedure is related to several tensor decomposition techniques, and the preferred mode is related to the tensor’s approximate multilinear rank [49]. Here, instead of decomposing a tensor across all modes we simply perform independent mode-1 and mode-2 decompositions and compare the quality of their corresponding reconstructions.

For the V1 dataset illustrated in **Fig 1**the neuron mode was preferred; it provided the least reconstruction error (**Fig 2C,** left). In contrast, for the M1 dataset illustrated in **Fig 1**the condition mode was preferred (**Fig 2C,** right). This analysis considered all time points in the shaded regions of **Fig 1**. Keeping in mind that reconstruction along either mode is expected to perform reasonably well (data points are rarely uncorrelated along any mode) the disparity between V1 and M1 is large: for V1 the basis-neuron reconstruction performed 33% better than the basis-condition reconstruction, while for M1 it performed 68% worse.

### The preferred mode emerges as more times are considered

**Fig 3:**
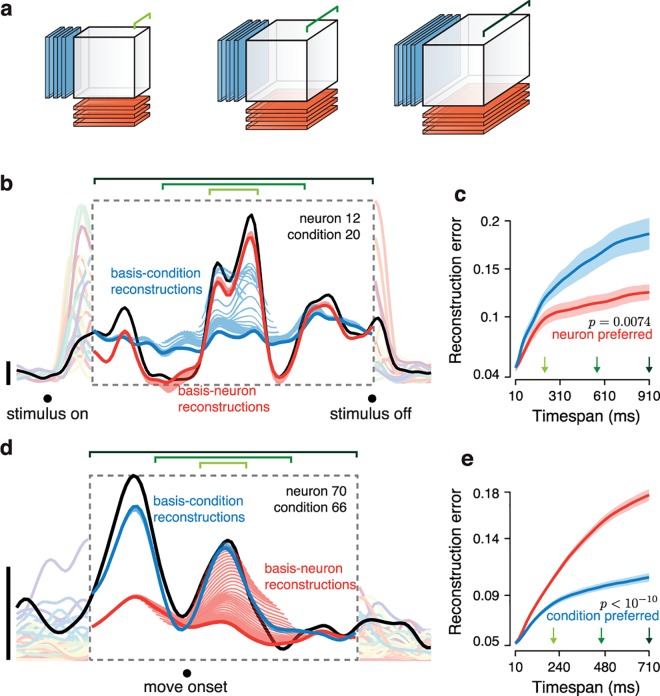
Illustration of the full preferred-mode analysis. Reconstruction error is measured as a function of the number of times included in the population tensor. (**a**) Schematic of the method. A fixed number (three in this simple illustration) of basis-neurons (red) and basis-conditions (blue) is used to reconstruct the population tensor. This operation is repeated for different subsets of time (*i*.*e*., different sizes of the population tensor) three of which are illustrated. Longer green brackets indicate longer timespans. (**b**) The firing rate (black) of one example V1 neuron for one condition, and its reconstruction using basis-neurons (red) and basis-conditions (blue). Short red/blue traces show reconstructions when the population tensor included short timespans. Longer red/blue traces show reconstructions when the population tensor was expanded to include longer timespans. Dark red/blue traces show reconstructions when the population tensor included all times. For illustration, data are shown for one example neuron and condition, after the analysis was applied to a population tensor that included all neurons and conditions (same V1 dataset as in **Figs 1A** and **2C**). The dashed box indicates the longest analyzed timespan. Responses of the example neuron for other conditions are shown in the background for context. Vertical bars: 10 spikes per second. (**c**) Plot of normalized reconstruction error (averaged across all neurons and conditions) for the V1 dataset analyzed in **b**. Red and blue traces respectively show reconstruction error when using 12 basis neurons and 12 basis conditions. The horizontal axis corresponds to the duration of the timespan being analyzed. Green arrows indicate timespans corresponding to the green brackets in **b**. Shaded regions show error bars (Methods). (**d**) As in **b** but illustrating the reconstruction error for one M1 neuron, drawn from the population analyzed in **Figs 1B** and **2C**. (**e**) As in **c** but for the M1 dataset, using 25 basis neurons and 25 basis conditions. The right-most values in **c** and **e** plot the reconstruction error when all times are used, and thus correspond exactly to the bar plots in **Fig 2C**.

A preferred mode exists because the population tensor spans multiple neurons, conditions, and times. Consider the population response at a single time, yielding an *N* × *C* × 1 subtensor (a matrix). For this case neither mode is preferred—the row rank (neuron mode) of a matrix equals the column rank (condition mode). How does the preferred mode emerge as more times are considered? We assessed reconstruction error as a function of timespan (**Fig 3**) beginning with a single time-point, halfway through the response. Using this time we chose bases of *k* elements such that there was a 5% reconstruction error of the *N* × *C* × 1 matrix (this determined the choice of *k* = 12 and 25 for the V1 and M1 datasets). Keeping *k* fixed, we increased the tensor size, adding both an earlier and a later time point (we considered time points sampled every 10 ms). Thus, reconstruction error was measured for subtensors of size *N* × *C* × *T*_*i*_ where *T*_*i*_ = 1,3,5,…,*T*.

The emergence of the preferred mode was often readily apparent even when reconstructing single-neuron responses (note that the entire tensor was always reconstructed, but each neuron can nevertheless be viewed individually). **Fig 3B** shows the response of one V1 neuron for one condition (black trace) with reconstructions provided by the neuron basis (red) and condition basis (blue). Each of the (shortened) light red and light blue traces show reconstructions for a particular timespan (*T*_*i*_). Dark red and dark blue traces show reconstructions for the full timespan (*T*_*i*_ = *T*). Unsurprisingly, for short timespans (short traces near the middle of the plot) the two reconstructions performed similarly: blue and red traces both approximated the black trace fairly well. However, for longer timespans the condition-mode reconstruction became inaccurate; the longest blue trace provides a poor approximation of the black trace. In contrast, the neuron-mode reconstruction remained accurate across the full range of times; short and long red traces overlap to the point of being indistinguishable. Thus, the reason why the V1 data were neuron-preferred (**Fig 2C**) is that the neuron basis, but not the condition basis, continued to provide good reconstructions across long timespans.

For the M1 dataset we observed the opposite effect (**Fig 3D**). For very short timespans both the neuron and condition bases provided adequate approximations to the black trace. However, for longer timespans the neuron-mode reconstruction (red) was unable to provide an accurate approximation. In contrast, the condition mode reconstruction remained accurate across all times; short and long blue traces overlap to the point of being indistinguishable.

The disparity in reconstruction error between the preferred and non-preferred mode was often clear at the single-neuron level, and was very clear at the population level. We computed overall reconstruction error for the population tensor as a function of timespan *T*_*i*_ (**Fig 3C and 3E**). The profile of each trace reflects reconstruction ‘stability.’ Reconstructions were never perfectly stable; error inevitably grew as more data had to be accounted for. However, stability was considerably better for the preferred mode: the neuron mode for V1 and the condition mode for M1. As can be inferred from the standard errors of the mean (shaded regions) reconstruction error in V1 was significantly lower for the neuron mode for all but the shortest windows (*p* = 0.007 for the longest window). Conversely, reconstruction error in M1 was significantly lower for the condition mode for all but the shortest windows (*p* < 10^−10^ for the longest window).

When a particular reconstruction fares poorly—*e*.*g*., the failure of the condition mode to accurately capture the firing rate of the V1 neuron in **Fig 3B**—it is not trivial to interpret the exact manner in which reconstruction failed. However, the underlying reason for poor reconstruction is simple: the data have more degrees of freedom along that mode than can be accounted for by the corresponding basis set. For V1, the data have more degrees of freedom across conditions than across neurons, while the opposite was true for M1.

Thus, different datasets can have strongly differing preferred modes, potentially suggesting difference sources of temporal response structure. Before considering this possibility, we ask whether the difference in preferred mode between V1 and M1 is robust, both in the sense of being reliable across datasets and in the sense of not being a trivial consequence of surface-level features of the data, such as frequency content, that differ between V1 and M1 recordings.

### Preferred-mode analysis of multiple datasets

**Fig 4:**
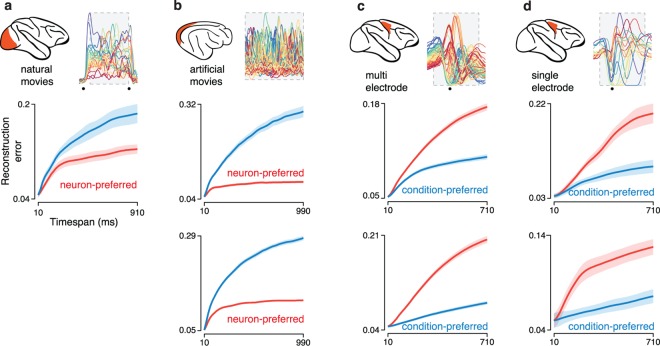
Preferred-mode analysis across neural populations. Each panel corresponds to a dataset type, and plots normalized reconstruction error as a function of timespan (as in **Fig 3C and 3E**). Excepting panel **a**, two datasets corresponding to two animals were analyzed, yielding two plots per panel. Insets at top indicate the dataset type and show the response of an example neuron. (**a**) Analysis for the V1 population from **Fig 1A**, recorded from a monkey viewing movies of natural scenes. Data are the same as in **Fig 3C** and are reproduced here for comparison with other datasets. (**b**) Analysis of two V1 populations recorded from two cats using grating sequences. (**c**) Analysis of two M1 populations (monkeys J and N) recorded using implanted electrode arrays. The top panel corresponds to the dataset illustrated in **Fig 1B** and reproduces the analysis from **Fig 3E**. (**d**) Analysis of two additional M1 populations from the same two monkeys but for a different set of reaches, with neural populations recorded sequentially using single electrodes.

To assess robustness we analyzed two additional V1 datasets recorded from cat V1 using 96-electrode arrays during presentation of high-contrast grating sequences[4,50] (**Fig 4B**; top, 50 different sequences; bottom 90 different sequences; panel **a** reproduces the analysis from **Fig 3C** for comparison). For all V1 datasets the neuron mode was preferred: reconstruction error grew less quickly with time when using basis-neurons (red below blue). We analyzed three additional M1 datasets (**Fig 4C and 4D**; the top of panel **c** reproduces the analysis from **Fig 3E** for comparison), recorded from two monkeys performing variants of the delayed reach task. For all M1 datasets the condition mode was preferred: reconstruction error grew less quickly with time when using basis-conditions (blue below red).

Most datasets involved simultaneous recordings (the three V1 datasets in **Fig 4A and 4B** and the two M1 datasets in **Fig 4C**). However, the preferred mode could also be readily inferred from populations built from sequential recordings (the two M1 datasets in **Fig 4D**). Critically, we note that sequential recordings employed the same stimuli for every neuron (stimuli were not tailored to individual neurons) and behavior was stable and repeatable across the time-period over which recordings were made.

To avoid the possibility that the preferred mode might be influenced by the relative number of recorded neurons versus conditions, all analyses were performed after down-selecting the data so that neuron count and condition count were matched (Methods). Typically, there were more neurons than conditions. We thus down-selected the former to match the latter. The preferred mode was, within the sizeable range we explored, invariant with respect to condition count. The three V1 datasets employed a different number of conditions (25, 90, and 50) yet all showed a neuron mode preference. The four M1 datasets employed a similarly broad range (72, 72, 18, and 18 conditions) yet all showed a condition mode preference. We further explored the potential impact of condition count by taking the 72-condition datasets in Fig 4C and restricting the number of analyzed conditions. The preferred mode was robust to this manipulation (see Methods) across the range tested (10–72 conditions). We also performed this analysis for all V1 datasets, and again found that the preferred mode was robust (not shown). Thus, even a modest number of conditions is sufficient to produce a clear preferred mode. That preferred mode then remains consistent as more conditions are added.

### The preferred mode is not related to surface-level features

Might the differing preferred modes in V1 and M1 be in some way due to differing surface-level features such as frequency content? *A priori* this is unlikely: properties such as frequency content may have an overall impact on the number of basis-set elements required to achieve a given accuracy, but there is no reason they should create a bias towards a particular preferred mode. Such a bias is also unlikely for three empirical reasons. First, as will be shown below, some existing models of M1 yield a condition-mode preference while others yield a neuron-mode preference. This occurs despite the fact that the surface-level structure produced by all such models resembles that of the M1 data. Second, the preferred mode remained unchanged when surface-level features were altered via temporal filtering (see Methods). In particular, V1 datasets remained neuron-preferred even when filtering yielded responses with lower frequency content than M1 responses. Third, it can be readily shown via construction that data with the surface-level features of V1 (or of M1) can have either preferred mode.

**Fig 5:**
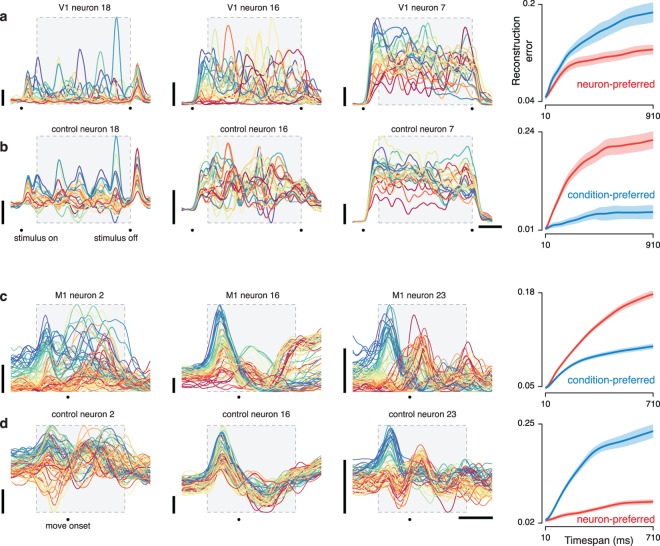
Preferred mode analysis of two control datasets. The preferred mode is not determined by surface-level features. (**a**) Analysis for the empirical V1 dataset from **Fig 3C** and **Fig 4A**. Shown are three example neurons (left panels) and reconstruction error versus timespan (right panel, reproduced from **Fig 3C**). (**b**) Same as in **a** but the V1 dataset was intentionally manipulated to have structure that was simplest across conditions. (**c**) Analysis for the empirical M1 dataset from **Fig 3E**. Shown are three example neurons (left panels) and reconstruction error versus timespan (right panel, reproduced from **Fig 3E**). (**d**) Same as in **c** but the M1 dataset was intentionally manipulated to have structure that was simplest across conditions.

To illustrate this last point we constructed data with the surface-level of features of V1 but with a condition-mode preference. We began with the V1 dataset analyzed in **Fig 4A** and extracted a set of ‘basis-conditions’ that captured most of the data variance. This was necessarily a large set of basis conditions (24) given the true neuron-mode preference of the data. We artificially reduced that number of basis conditions by summing random sets of the original basis conditions. For example, the new first basis condition might be a sum of the original basis conditions 1, 7, 12 and 23. Thus, the same patterns were present in the data (no basis conditions were removed) but the degrees of freedom were greatly reduced. We then constructed an artificial population response by replacing the original response of each neuron with the linear combination of modified basis conditions that best approximated the original response. This manipulation resulted in a control dataset with responses that are intentionally altered yet retain the surface-level features of the original data (**Fig 5A,** original data; **Fig 5B**, control data). The manipulated V1 data had a strong condition-mode preference, (blue lower than red) in opposition to the true neuron-mode preference of the original data. Using the same procedure (but reducing degrees of freedom within the neuron basis) we constructed control M1 datasets where surface-level features were preserved but where the neuron mode became preferred (**Fig 5D**, red lower than blue) in opposition to the original data (**Fig 5C**, top, blue lower than red). Thus, the preferred mode is not a consequence of surface-level features.

### The preferred mode of simulated M1 populations reflects model class

We were interested in the possibility that the origin of temporal structure might influence the preferred mode. Specifically, tuning for external variables might constrain structure across neurons; if responses reflect a fixed number of external variables then neurons would be limited to that many degrees of freedom. Conversely, internal dynamics might constrain structure across conditions; if each condition evolves according to the same dynamics, conditions could differ along limited degrees of freedom.

The above intuition agrees with the neuron-preferred tensor structure of the V1 datasets, for which the trial-averaged response is expected to be dominated by the stimulus-driven component. Does this intuition extend to, and perhaps help differentiate, models of M1? Many prior studies have modeled M1 responses in terms of tuning for of movement parameters (target direction, reach kinematics, joint torques, etc.). Although the causality is assumed to be reversed relative to V1 (with the M1 representation producing the downstream kinematics), such models formally treat neural responses as functions of time-varying external variables; in particular, responses differ across neurons because different neurons have different tuning for those external variables. M1 ‘tuning-based models’ are thus fundamentally similar to tuning models of V1. On the other hand, some recent studies have modeled M1 responses as the outcome of internal population level dynamics that are similar across conditions. In such models, downstream quantities such as muscle activity are assumed to be a function of cortical activity but cortical activity is not a function of downstream quantities (due to non-invertibility). These M1 ‘dynamics-based models’ are thus fundamentally dissimilar from tuning models of V1.

**Fig 6:**
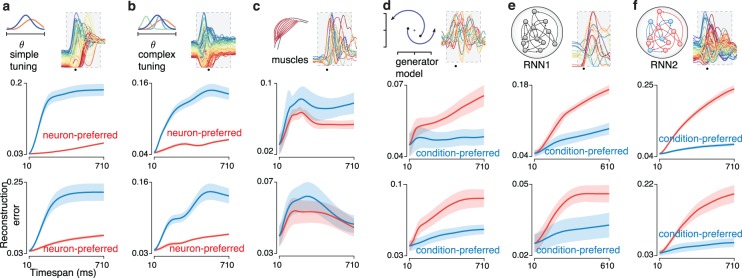
Preferred-mode analysis for non-neural data. Analysis is shown for ten simulated datasets and two muscle populations. Presentation as in **Fig 4**. (**a**) Analysis of simulated M1 populations from the simple tuning model. Two simulated populations (top and bottom) were based on recorded kinematic parameters of two animals (J and N), acquired during the same experimental sessions for which the neural populations are analyzed in **Fig 4C**. (**b**) As in **a**, but M1 populations were simulated based on a more complex tuning model. (**c**) Analysis of populations of muscle responses (monkeys J and N, top and bottom) recorded using the same task/conditions as in **Fig 4D**. (**d**) Analysis of two simulated M1 populations from the dynamical ‘generator model’ that was trained to reproduce patterns of muscle activity. The model was trained to produce the patterns of deltoid activity from the muscle populations in panel **c**. (**e**) Analysis of two simulated M1 populations from a neural network model trained to produce the patterns of muscle activity shown in panel **c**. (**f**) Analysis of two simulated M1 populations from a ‘non-normal’ neural network model.

We analyzed simulated data from five published models of M1, including two models based on tuning for kinematic variables [30] and three models that assumed strong population-level dynamics subserving the production of muscle activity [30,34,36]. All M1 models displayed surface-level features that resembled those of the recorded M1 responses, including a burst of multiphasic responses. Each simulated dataset had neuron and condition counts matched with a corresponding neural population. Each model was simulated twice (top and bottom of the relevant panels in **Fig 6A, 6B, 6D, 6E and 6F**) with each instance being based on the empirical kinematics or muscle activity for one of the neural datasets.

The neuron mode was preferred for the two models that were based on tuning for kinematics (**Fig 6A and 6B** red below blue). For the first tuning-based model (**Fig 6A**), the relevant kinematic variables were hand velocity and speed (the magnitude of velocity) as in [51]. For the second tuning-based model (**Fig 6B**), the kinematic variables also included hand position and acceleration [52]. Thus, the second tuning-based model reflects the possibility that neural responses are complex due to tuning for multiple movement-related parameters—a position which has recently been argued for based on the ability to decode such parameters [46].

The condition mode was preferred for the three models (**Fig 6D, 6E and 6F**) that employed strong population-level dynamics. The model in **Fig 6D** was based on a pair of simple oscillations that followed approximately linear dynamics and provided a basis for fitting empirical patterns of muscle activity [30]. The model in **Fig 6E** was a nonlinear recurrent neural network (RNN) trained to produce the empirical muscle activity patterns [34]. The model in **Fig 6F** was an RNN with ‘non-normal’ dynamics realized via separate excitatory and inhibitory populations[36]. Critically, these three dynamics-based models were not fit to neural responses; their responses reflect the dynamics necessary to produce the desired outputs. Each has been recently proposed as a possible model of M1 activity during reaches. Despite their substantial architectural differences, all dynamics-based models displayed a condition-mode preference (blue below red).

In a subsequent section we employ a formal approach to explore why different model classes produce different preferred modes. Presently, we simply stress that the preferred mode can be used to test model predictions. In particular, the tuning-based models displayed neuron-preferred tensor structure in opposition to the data. In contrast, the dynamics-based models displayed condition-preferred tensor structure in agreement with the data. Thus, although all models of M1 reproduced (to some reasonable degree) the basic surface-level features of M1 responses, only the dynamics-based models predicted the true condition-mode preference of the M1 population data.

We also analyzed the tensor structure of populations of recorded muscles. Because muscle activity is in some sense an external movement parameter, one might expect the muscle population to be neuron-preferred, in agreement with the tuning-based models above. On the other hand, the dynamics-based models were trained so that a linear projection of the model population response replicated the empirical muscle population response. Given this tight link one might expect the muscle population be condition-preferred. Empirically, the muscle populations had no clear preferred mode: reconstruction error was similar and in some cases overlapping for the neuron and condition modes. There was an overall tendency for the muscle data to be neuron-preferred (the blue trace tended to be above the red trace at many points) but this was not statistically compelling (*p* = 0.37 and *p* = 0.80).

This analysis of muscle populations again highlights that the preferred mode cannot be inferred from surface-level features. Muscle responses and neural responses share many similar features yet do not show the same tensor structure. The muscle data also highlight that a clear preferred mode need not exist for all datasets. Furthermore, the tensor structure of a system’s outputs need not reflect the tensor structure of the system itself. Dynamics-based models built to produce muscle activity showed robust condition-mode preferences (**Fig 6D, 6E and 6F**). Yet the muscle populations themselves did not show a condition mode preference (if anything they were weakly neuron-preferred). We return later to the point that the output of a dynamical system need not share the same preferred mode as the system itself.

As a side note, a natural desire is to examine the bases themselves, which might be informative regarding the underlying model. For example, the first basis neuron is essentially the projection of the data onto the first principle component of the *N* × *N* covariance matrix that captures covariance between neurons. The first basis condition is the same, but for a *C* × *C* covariance matrix that captures covariance between conditions. It is indeed possible to make inferences from both such projections [29,30], yet this typically requires specific hypotheses and tailored analysis methods. The fundamental hurdle is that, for any given basis set, there are infinitely many rotations of that basis set that provide equally good reconstruction. Thus, the details of any given projection can be difficult to interpret without bringing additional information to bear. We therefore focus in this study on the quality of the reconstruction, rather than the features of the basis set.

### The preferred mode is robust to the number of basis elements

We assessed whether the preferred mode analysis is robust to a key parameter: the number of basis-elements used when quantifying reconstruction error. This is important because it is not possible to directly measure the degrees of freedom (*i*.*e*., the number of basis elements that produces zero reconstruction error) for each mode, given measurement noise and other practical considerations. For this reason, the analyses above compared not degrees of freedom *per se*, but rather the reconstruction error for a fixed number of degrees of freedom. Before concluding that data have fewer degrees of freedom across one mode versus another, one should assess whether the preferred mode is robust with respect to the choice of that fixed number.

**Fig 7:**
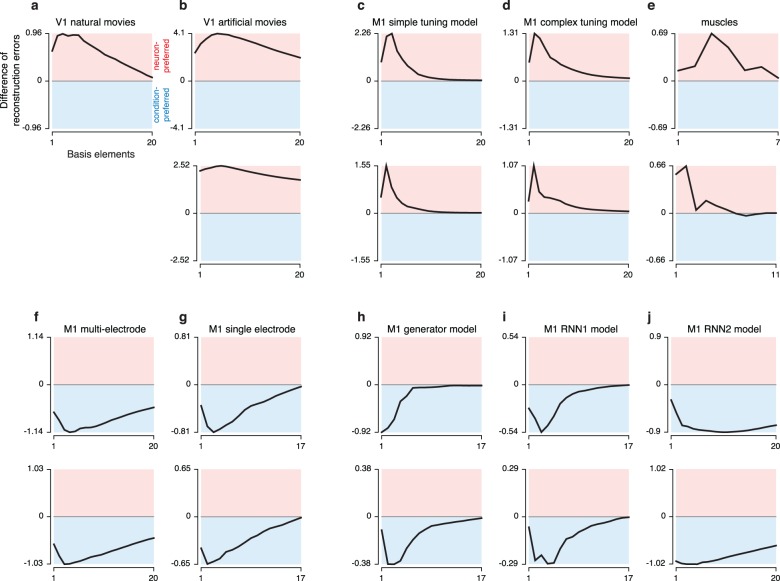
Reconstruction error as a function of the number of basis elements. Each panel plots the difference in reconstruction errors (reconstruction error using *k* basis-conditions minus reconstruction error using *k* basis-neurons). The full timespan is considered. Positive values indicate neuron-preferred structure while negative values indicate condition-preferred structure (colored backgrounds for reference). All values in each panel are normalized by a constant, chosen as the smaller of the two reconstruction errors (for the full timespan) plotted in corresponding panels of **Figs 4**and **6**. For most datasets we considered *k* from 1–20 (mode preference did not flip for higher *k* in any dataset). For datasets with fewer than 20 neurons (or muscles) values are plotted up to the maximum possible *k*: the number of neurons (or muscles) in the dataset.

To assess robustness we focused on the difference in error between the condition-mode reconstruction and the neuron-mode reconstruction for the longest time window (*T*_*i*_ = *T*). We swept the number of basis elements and plotted the normalized difference in reconstruction errors (**Fig 7**). Positive values indicate a neuron-mode preference and negative values indicate a condition-mode preference. We considered from 1–20 basis elements, stopping earlier if the dataset contained fewer than 20 total degrees of freedom (e.g., the M1 single-electrode data had 18 conditions and the muscle populations contained 8 and 12 recordings respectively). All datasets displayed a preferred mode that was robust with respect to the number of basis elements. In most cases the preferred mode was clearest when a modest number of basis elements was used. Indeed, there was often a peak (for neuron-preferred datasets; data lying in the red shaded area) or trough (for condition-preferred datasets; data lying in the blue shaded area). Unsurprisingly, the difference in reconstruction error trended towards zero as the number of basis elements became large (the difference is necessarily zero if the number of basis elements is equal to the number of neurons / conditions in the data itself).

The analysis in **Fig 7**supports the results in **Figs 4**and **6**. All V1 datasets and all M1 tuning-model datasets were consistently neuron-preferred. All M1 datasets and all dynamical M1 models were consistently condition-preferred. The muscle populations, which had trended weakly towards being neuron-preferred in the analysis in **Fig 6**, trended more strongly in that direction when examined across reconstructions based on different numbers of basis elements (**Fig 7E**). Thus, if a dataset had a clear preference for our original choice of basis elements (the number necessary to provide a reconstruction error <5% when using a single time-point) then that preference was maintained across different choices, and could even become stronger. The analysis in **Fig 7**also underscores the very different tensor structure displayed by different models of M1. Dynamics-based models (panels **h,i,j**) exhibited negative peaks (in agreement with the empirical M1 data) while tuning-based models (panels **c,d**) and muscle activity itself (panel **e**) exhibited positive peaks.

### Possible sources of tensor structure

Why did tuning-based models display a neuron-mode preference while dynamics-based models displayed a condition-mode preference? Is there formal justification for the motivating intuition that the origin of temporal response structure influences the preferred mode? This issue is difficult to address in full generality: the space of relevant models is large and includes models that contain mixtures of tuning and dynamic elements. Nevertheless, given reasonable assumptions—in particular that the relevant external variables do not themselves obey a single dynamical system across conditions—we prove that the population response will indeed be neuron-preferred for models of the form:

x(t,c)=Bu(t,c), (4)

where *x* ∈ ℝ^*N*^ is the response of a population of *N* neurons, *u* ∈ ℝ^*M*^ is a vector of *M* external variables, and *B* ∈ ℝ^*N*×*M*^ defines the mapping from external variables to neural responses. The *n*th row of *B* describes the dependence of neuron *n* on the external variables *u*. Thus, the rows of *B* are the tuning functions or receptive fields of each neuron. Both *x* and *u* may vary with time *t* and experimental condition *c*.

A formal proof, along with sufficient conditions, is given in Methods. Briefly, under Eq (4), neurons are different views of the same underlying *M* external variables. That is, each *u*_*m*_(*t*,*c*) is a pattern of activity (across times and conditions) and each *x*_*n*_(*t*,*c*) is a linear combination of those patterns. The population tensor generated by Eq (4) can thus be built from a linear combination of *M* basis-neurons. Critically, this fact does not change as time is added to the population tensor. Eq (4) imposes no similar constraints across conditions; *e*.*g*., *u*(:,*c*_1_) need not bear any particular relationship to *u*(:,*c*_2_). Thus, a large number of basis-conditions may be required to approximate the population tensor. Furthermore, the number of basis-conditions required will typically increase with time; when more times are considered there are more ways in which conditions can differ. A linear tuning model therefore implies a neuron-mode reconstruction that is stable with time and a condition-mode reconstruction that is less accurate and less stable.

Conversely, the population response will not be neuron-preferred (and will typically be condition-preferred) for models of the form:

x(t+1,c)=Ax(t,c), (5)

Where *A* ∈ ℝ^*N*×*N*^ defines the linear dynamics. This equation admits the solution *x*(*t*,*c*) = *A*^*t*−1^*x*(1,*c*). Thus, the matrix *A* and the initial state *x*(1,*c*) fully determine the firing rate of all *N* neurons for all *T* times. In particular, the linear dynamics captured by *A* define a set of *N* × *T* population-level patterns (basis-conditions) from which the response for any condition can be built via linear combination. Critically, this fact does not change as different timespans (*T*_*i*_) are considered. Although the size of each *N* × *T*_*i*_ basis-condition increases as *T*_*i*_ increases, the number of basis-conditions does not. In contrast, the number of necessary basis-neurons may grow with time; neural activity evolves in some subspace of ℝ^*N*^ and as time increases activity may more thoroughly explore this space. Thus, a linear dynamical model implies a condition-mode reconstruction that is stable with time, and a neuron-mode reconstruction that is less accurate and less stable (for proof see Methods).

The above considerations likely explain why we found that tuning-based models were always neuron-preferred and dynamics-based models were always condition-preferred. While none of the tested models were linear and some included noise, their tensor structure was nevertheless shaped by the same factors that shape the tensor structure of more idealized models.

### The preferred mode in simple models

Tuning-based models and dynamics-based models are extremes of a continuum: most real neural populations likely contain some contribution from both external variables and internal dynamics. We therefore explored the behavior of the preferred mode in simple linear models where responses were either fully determined by inputs, were fully determined by population dynamics, or were determined by a combination of the two according to:

x(t+1,c)=Ax(t,c)+Bu(t,c). (6)

**Fig 8:**
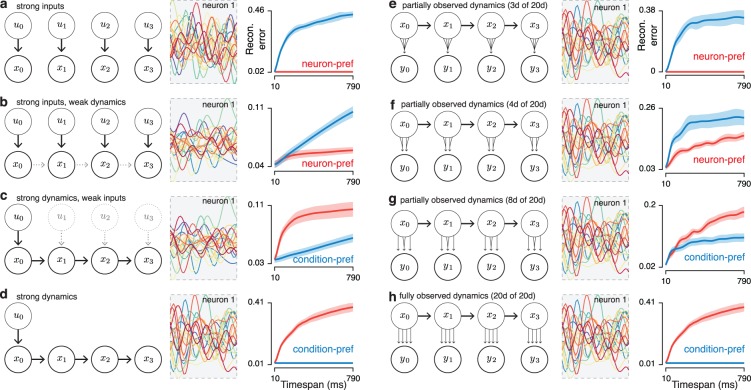
The preferred-mode analysis applied to simulated linear dynamical systems. Left column of each panel: graphical models corresponding to the different systems. Middle column of each panel: response of neuron 1 in each simulated dataset. Colored traces correspond to different conditions. Right column of each panel: preferred-mode analysis applied to simulated data from that system. Analysis is performed on the data *x* in panels **a-d**, while analysis is performed on the data *y* in panels **e-h**. (**a**) A system where inputs *u* are strong and there are no internal dynamics (*i*.*e*., there is no influence of *x*_*t*_ on *x*_*t*+1_. (**b**) A system with strong inputs and weak dynamics. (**c**) A system with weak inputs and strong dynamics. (**d**) A system with strong dynamics and no inputs other than an input *u*_0_ at time zero that sets the initial state. (**e**) A system with 20-dimensional linear dynamics at the level of the state *x*, but where the observed neural responses *y* reflect only 3 of those dimensions. *I*.*e*., the linear function from the state *x* to the neural recordings *y* is rank 3. (**f**) A system with 20-dimensional dynamics and 4 observed dimensions. (**g**) A system with 20-dimensional dynamics and 8 observed dimensions. (**h**) A system with 20-dimensional dynamics where all 20 dimensions are observed (formally equivalent to the case in panel **d**).

The case where responses are fully determined by inputs is formally identical to a tuning model; inputs can be thought of either as sensory, or as higher-level variables that are being represented by the population. When *A* was set to 0 and responses were fully determined by inputs (**Fig 8A**) the neuron mode was preferred as expected given the formal considerations discussed above. Indeed, because the model is linear, neuron-mode reconstruction error was perfectly stable as times were added (the red trace remains flat). When *B* was set to zero and responses were fully determined by internal dynamics acting on an initial state, the condition mode was preferred and condition-mode reconstruction error was perfectly stable (**Fig 8D**), consistent with formal considerations.

For models where tuning for inputs was strong relative to dynamics, the neuron mode was preferred (**Fig 8B**). However, because dynamics exerted a modest influence, neuron-mode reconstruction error was not perfectly stable. When dynamics were strong relative to inputs, the condition mode was preferred (**Fig 8C**). However, because inputs exerted a modest influence, condition-mode reconstruction error was not perfectly stable. Thus, simple simulations confirm the expected behavior. A neuron-mode preference is produced when temporal response structure is dominated by tuning for inputs, even if dynamics exert some influence. A condition-mode preference is produced when temporal response structure is dominated by dynamics, even if inputs exert some influence. Thus, the preferred-mode analysis can reveal the dominant source of structure, but does not rule out other contributions.

A potentially confusing point of interpretation is that all neurons necessarily respond to inputs; each neuron is driven by the inputs it receives. How then can there be a difference in tensor structure between a population that is tuned for inputs versus a population that reflects dynamics? The answer lies in how fully the population reflects dynamics. In the case of tuning for external variables, those variables typically do not fully reflect dynamics. Although the local environment is in some sense ‘dynamic,’ those dynamics are incompletely observed via the sensory information available to the nervous system. Conversely, if dynamics are produced by the local population they may be fully observed provided that sufficient neurons are recorded.

To illustrate this point we repeated the simulations with the model population either partially (**Fig 8E**) or completely (**Fig 8H**) reflecting an identical set of underlying dynamics. As expected, the case where dynamics are partially observed behaved like the case when the system is input driven: the neuron mode was preferred. As dynamics became more fully reflected, the population switched to being condition-preferred. Thus, condition-preferred structure results from a very particular circumstance: the neural population obeys dynamics that are consistent across conditions and are close to fully reflected in the neural population itself. In contrast, neuron-preferred structure is observed when the temporal structure is inherited from outside the system: from sensory inputs or from dynamics that may be unfolding elsewhere in the nervous system. This explains why there is no paradox in the fact that the muscle populations tended to show neuron-preferred structure (**Fig 6C and Fig 7E**) even though dynamical models that produce muscle activity show condition-preferred structure (**Fig 6D–6F, Fig 7H–7J**) as does M1 itself. More generally, these simulations illustrate that one may often expect a difference in preferred mode between a system that produces a motor output and a system that ‘listens’ to that output (*e*.*g*., a sensory system that provides feedback during movement).

A key point illustrated by the simulations in **Fig 8A–8D** is that the preferred mode is independent of smoothness in the temporal domain. For example, the idealized models in **Fig 8A** and **8D** have responses with closely matched temporal smoothness, yet yield opposing preferred modes. This can be understood via reference to the derivation in the Methods, where assumptions regarding temporal smoothness play no role. For example, a condition-mode preference will be observed even if dynamics cause rapid fluctuations in the neural state, and indeed even if the dynamics are themselves rapidly time-varying. It is the ‘smoothness’ across conditions versus neurons that determines the preferred mode, not the smoothness across time. This fact is also illustrated in **Fig 5**, where control manipulations alter the preferred mode while leaving temporal smoothness unchanged.

For the simulations in **Fig 8**and the models in **Fig 6**the preferred mode always reflected the dominant source of temporal structure. Yet with the exception of some idealized models, reconstruction error was rarely perfectly stable even for the preferred mode. The lack of perfectly stability arises from multiple sources including nonlinearities, simulated noise in the firing rate, and contributions by the non-dominant source of structure. We therefore stress that it is difficult, for a given empirical dataset, to ascertain why the preferred mode shows some instability in reconstruction error. For example, in the case of M1 it is likely that the modest rise in condition-mode reconstruction error with timespan (*e*.*g*., **Fig 4C and 4D**) reflects all the above factors.

## Discussion

Our analyses were motivated by three hypotheses: first, that population responses will show tensor structure that deviates strongly from random, being simpler across one mode than another; second, that the ‘preferred mode’ will likely differ across datasets; and third, that the underlying source of temporal response structure influences the preferred mode. The empirical data did indeed deviate strongly from random. V1 datasets were consistently neuron-preferred: the population response was most accurately reconstructed using basis-neurons. M1 datasets were consistently condition-preferred: the population response was most accurately reconstructed using basis-conditions. This difference was invisible at the single-neuron level and could not be inferred from surface-level features of the data. Simulations and formal considerations revealed that neuron-preferred structure arises preferentially in models where responses reflect stimuli or experimental variables. Condition-preferred tensor structure arises preferentially in models where responses reflect population-level dynamics.

### Implications for models of motor cortex responses

Given the relationship between model class and preferred mode, the neuron-preferred structure in V1 is entirely expected: all V1 datasets were recorded in the presence of strong visual inputs that are expected to drive the observed response structure [53]. In contrast, the condition-preferred structure of the M1 population response could not be anticipated from first principles because there is little agreement regarding the source of temporal response structure in M1. Several existing M1 models assume that time-varying responses are a function of time-varying movement variables such as reach direction, velocity, and joint torques (for a review see [21]). These variables may be ‘dynamic’ in the loose sense (they change with time and some may be derivatives of the others) but their values typically do not follow a single dynamical rule that is consistent across conditions. Other recent models are explicitly dynamics-based: the future population state is a function of the present population state, with external inputs serving primarily to set the initial state of the dynamics [30,34,36]. Tuning-based and dynamics-based models lie on a continuum, but occupy opposing ends and thus make different predictions regarding the tensor structure of the population response. Existing dynamics-based models predict condition-preferred tensor structure, in agreement with the M1 data. Existing tuning-based models predict neuron-preferred structure, in opposition to the M1 data.

Our results thus place strong constraints on models of M1: to be plausible a model must replicate the condition-preferred structure of the empirical population response. Our exploration of current models indicates that this happens naturally for models that include strong dynamics within the recorded population. It does not occur naturally for tuning-based models. We cannot rule out the possibility that future elaborations of tuning-based models might be able to replicate the empirical condition-preferred structure, but the practical possibility of such elaborations remains unclear. There also exist a number of M1 models that we did not examine [35,37,54,55]. It remains an empirical question whether the tensor structure of such models is compatible with the data.

We stress that all current M1 models (including those that successfully predict the empirical preferred mode) are incomplete in key ways and will need to be elaborated or unified in the future. For example, the dynamics-based models we examined do not yet capture the influence of external, sensory-based feedback which is known to be a driver of M1 responses [38,39,56]. Conversely, a recent model of feedback control (not tested here) captures only the dynamics of external feedback loops; the M1 population was modeled as a feedforward network [37]. As future models are developed that incorporate both internal recurrence and sensory feedback, tensor structure provides a simple test regarding whether those models produce realistic population-level responses.

Tensor structure is a basic feature of data, much as the frequency spectrum or the eigenvalue spectrum of the neural covariance matrix are basic features of data. (Indeed, tensor structure is a simple extension to a three-mode array of the standard method of applying principal component analysis to a two-mode array.) Thus, any model that attempts to explain data should succeed in replicating the preferred mode. This requirement is particularly important because, while models can often be easily modified to produce obvious surface-level features, it is more challenging to also reproduce the underlying tensor structure. Just as importantly, the preferred mode of recorded data can be informative regarding how an appropriate model should be constructed. For every model tested we found that tensor structure is condition-preferred only when the measured population reflects most of the state variables in a dynamical system. In the context of M1, this suggests that successful models will be those where a large percentage of the relevant state variables (sensory feedback, muscle commands and the dynamics that link them) are observable in the M1 population response.

It should be stressed the preferred mode is likely not a feature of a brain area *per se*, but rather of a neural population in the context of the computation being performed by that population. For example, M1 has strong responses to sensory stimuli, especially stretching of the tendons and muscles [56]. In an experiment where responses are driven primarily by externally imposed perturbations of the arm [57,58] it seems likely that M1 would exhibit a neuron-mode structure like that of V1 in the present study. If so, then it would be natural to apply a model in which responses are largely externally driven. If not, then one would be motivated to consider models in which external events set in motion internal dynamics. In either case, knowing the preferred mode would be valuable because it would constrain the set of plausible models.

### Interpretational caveats

Interpretation of the preferred mode is most straightforward when there exists one or more models that seek to explain the data. Any model (or model class) that does not replicate the empirical preferred mode must be modified or discarded. Can similarly strong inferences be drawn directly from the preferred mode of the data, without comparison with models? In short they cannot: while a robust preferred mode may suggest a particular class of model, caveats apply. As shown in the derivation (Methods) idealized models produce neuron-preferred structure when responses are driven by unconstrained external variables, and condition-preferred structure when responses are shaped by internal dynamics. We found that this pattern was robust under less-idealized circumstances: all of the models we examined exhibited a preferred mode consistent with the idealized pattern, even though they departed from idealized assumptions (in particular they were not linear). Such robustness is largely expected. For example, non-linear dynamical systems can often be well approximated by time-varying linear systems, which is all that is required to produce the idealized pattern. Similarly, a non-linear dependency on external variables can often be reconceived as a linear dependency via a change in variables.

That said, there will be limits to the observed robustness. It is possible that a model of one class (*e*.*g*., a dynamical systems model) can produce a paradoxical preferred mode (*e*.*g*., a neuron-mode preference) under certain circumstances. This might, for example, occur for a neural circuit with strongly nonlinear dynamics that produces long motor sequences. Such a system might be poorly approximated by time-varying linear dynamics, which would result in compromised condition-mode reconstructions. In the case where responses are driven by external variables, an unclear or even paradoxical preferred mode could occur if there is something ‘ill-conditioned’ about the input. For example, the input could be highly redundant across conditions, resulting in responses that lack enough structure to allow meaningful comparison of reconstruction quality for the neuron mode versus the condition mode. Along similar lines, it would be difficult to interpret the preferred mode in the case where there is little variation in the motor output that can be captured across conditions.

An attractive feature of the preferred mode analysis is that it can be applied without knowledge of the inputs to a system, and provides constraints on potential hypotheses without requiring fully mature models that are ready to be fit directly to data. These advantages are large but, as discussed above, not absolute. First, although potential inputs need not be known, one must have reasonable confidence that the task evokes a range of reasonably rich responses, such that a clear preferred mode can emerge. Second, interpretation of the preferred mode will always be most certain in the case where the preferred mode of the data can be compared with the preferred mode displayed by competing models. In the present case, the preferred mode of the M1 datasets consistently disagreed with the preferred mode of models where time-varying responses are a function of time-varying movement variables. As this accords with formal expectations, such models are unlikely to provide a good account of the data without major modification.

### Future applications

It is likely that neural populations outside of areas V1 and M1 will also display clear preferred modes, which could be diagnostic regarding candidate models. Applicable datasets are those that are sufficiently rich: the experimental task must elicit time-varying responses where PSTHs vary across neurons and conditions. Further, there must be sufficiently many neurons and conditions such that certain low-rank conditions are met (an explanation of these conditions are in Methods under Low-rank assumptions).

As a potential example, some models of decision-making assume that neural responses reflect a small number of task variables (*e*.*g*., a ‘decision variable’ whose value codes the evolving tendency towards a given choice [59]). Other models include internal dynamics that implicitly gate when information is integrated or ignored [60]. None of these decision models sits fully at an extreme—all assume both sensory inputs and some form of integration—but they possess large qualitative differences that may predict different tensor structure. Given the ease with which the preferred mode can be computed for both real and simulated data, the preferred-mode analysis provides a natural way to test whether a given model matches the data at a basic structural level.

## Methods

### Ethics

All methods were approved in advance by the respective Institutional Animal Care and Use Committees at Albert Einstein College of Medicine (protocol #20150303) and the New York State Psychiatric Institute (protocol #1361). To minimize any potential suffering non-survival surgeries were performed under deep anesthesia with sufentanil citrate, adjusted per the needs of each animal. Survival surgeries were performed under isoflurane anesthesia with carefully monitored post-operative analgesia.

### Experimental datasets

We analyzed 9 physiological datasets. Eight have been analyzed previously and one was recorded for the present study. All datasets were based on the spiking activity of a neural population recorded using either multi-electrode arrays (the datasets analyzed in **Fig 4A, 4B and 4C**) or sequential individual recordings (the neural dataset analyzed **Fig 4D** and the muscle dataset analyzed in **Fig 6C**). Datasets are available from the Dryad repository (http://dx.doi.org/10.5061/dryad.92h5d).

One V1 dataset (analyzed in **Figs 1, 2, 3, 4A** and **7A**) was collected using natural-movie stimuli from an anaesthetized adult monkey (*Macaca fascicularis*) implanted with a 96-electrode silicon ‘Utah’ array (Blackrock Microsystems, Salt Lake City, UT) in left-hemisphere V1. These data were recorded in the laboratory of Adam Kohn (Albert Einstein College of Medicine) specifically for the present study. The left eye was covered. Receptive field centers (2–4 degrees eccentric) were determined via brief presentations of small drifting gratings. Stimuli, which spanned the receptive fields, were 48 natural movie clips (selected from YouTube) with 50 repeats each. The frame rate was 95 Hz. Each stimulus lasted 2.63 s (100 movie frames followed by 150 blank frames). Spikes from the array were sorted offline using MKsort (available at https://github.com/ripple-neuro/mksort/). Single units and stable multi-unit isolations were included. Some neurons showed weak responses and were not analyzed further. Similarly, some stimuli (*e*.*g*., those where the region within the receptive fields was blank or relatively unchanging) evoked weak responses overall. Again, these were not analyzed further. Finally, to ensure we were analyzing a neural population that responds to a shared set of stimulus features, all analyses focused on the subset of units with strongly overlapping receptive fields, defined as the 25 units with receptive fields closest to the center of the stimulus. We insisted upon this criterion because our central analyses would not be as readily interpretable if applied to a set of neurons with distant receptive fields, as they would effectively be responding to different stimuli.

We analyzed two further V1 datasets (**Fig 4B**) recorded from cat V1 as described in [4,50] using Utah arrays implanted so as to overlap areas 17 and 18 (collectively, cat area V1). Stimuli were large stationary gratings, ~30 deg in diameter, and thus spanned the receptive fields of all neurons. Gratings were presented in a rapid sequence—one every 32 ms—each with one of 4 spatial phases and one of 12 orientations. One dataset had five sequences of ~12 s in length. The other dataset had nine such sequences. We wished to segment these long-duration stimuli into ‘conditions’ with a timescale comparable to that of the other V1 and M1 datasets analyzed here. To do so, we divided the first 10 s of each sequence into 10 one-second segments, which we treated as separate conditions (the stimuli in each second were unrelated to the stimuli in the last second, and are thus effectively different conditions). The two datasets (**Fig 4B**, top, bottom) thus yielded a total of 50 and 90 conditions, respectively. Each condition was observed across multiple (~10) trials. Each dataset consisted of 96 well-tuned multiunit recordings (see [4,50] for details), which were down-selected to match condition counts (50 and 90) of the datasets.

Four M1 datasets were recorded from two male macaque monkeys (*Macaca mulatta*) trained to perform a delayed reach task. These datasets have been described and analyzed previously [29,30]. Briefly, reaches were performed on a fronto-parallel screen for juice reward. To begin each trial the monkey touched a central spot. After a >400 ms hold period, a reach target and up to nine ‘barriers’ appeared (see **Fig 1** of [29]). The monkey was required to hold its position for a 0–1000 ms delay until a ‘go cue’, and to then briskly reach to the target while avoiding the barriers. A juice reward was delivered after a 450 ms hold period. This task evoked a large variety of conditions: each corresponding to a particular target and arrangement of barriers. For a given condition, reach trajectories were highly stereotyped across trials (there was only one allowable route through the barriers) allowing a meaningful computation of the average across-trial firing rate. Only trials with delays >450 ms were analyzed (5–40 trials per condition, depending on the dataset); shorter delays simply provided incentive to prepare their movement during the delay. For present purposes, the primary value of the barriers was that they increased the variety of reach conditions, thus increasing the size of the tensor that could be analyzed. In the original dataset some conditions included ‘distractor’ targets that the monkey had to ignore while preparing the reach. The purpose of those conditions was incidental to the present study and they were not included in the analysis (results were virtually identical if they were included). Neural responses were recorded from M1 and the adjacent region of caudal PMd. Single-electrode and array datasets employed 18 and 72 conditions respectively. Single-electrode datasets consisted of ideally isolated single neurons. Array datasets included both ideal isolations and good multi-unit isolations (*e*.*g*., two clear units that could not be separated from one another). Unit counts for the four datasets were 170, 218, 55, and 118 (corresponding, respectively, to panels **c-d** in **Fig 4**), which were down-selected to 72, 72, 18, and 18 to match condition counts.

Two datasets of the responses of muscle populations (analyzed in **Fig 6C**) were recorded using the same monkeys and task as for the M1 datasets. Muscle datasets used the same 18 conditions as the single-electrode datasets. EMG responses were recorded percutaneously using electrodes inserted for the duration of the recording session. Recordings were made from six muscle groups: deltoid, biceps brachii, triceps brachii, trapezius, latissimus dorsi and pectoralis. Multiple recordings were often made from a given muscle (*e*.*g*., from the anterior, lateral and posterior deltoid). For monkey J the triceps was minimally active and was not recorded. Muscles were recorded sequentially and then analyzed as a population (just as were the single-electrode datasets). For the two monkeys the resulting populations consisted of 8 and 12 recordings.

### Model datasets

We analyzed multiple datasets produced via simulation of published models. The velocity model from [30] was analyzed in **Fig 6A** (here, referred to as the simple tuning model). The complex-kinematic model from [30] was analyzed in **Fig 6B** (here referred to as the complex tuning model). The generator model from [30] is analyzed in **Fig 6D**. The network model of Sussillo et al. [34] is analyzed in **Fig 6E**. The network model of Hennequin et al. [36] is analyzed in **Fig 6F**. Both network models are instantiations of a recurrent neural network (RNN):

$$\frac{dx(t,c)}{dt}=-x(t,c)+Ar(t,c)+Bu(t,c)$$ (7)

r(t,c)=ϕ(x(t,c))

y(t,c)=Wr(t,c),

where *x* ∈ ℝ^*N*^ is the network state, *u* ∈ ℝ^*M*^ is the vector of inputs, *y* ∈ ℝ^*P*^ is the vector of outputs. The function *ϕ* is an element-wise nonlinear function, *r* ∈ ℝ^*N*^ is interpreted as a firing rate, and the matrices *A*, *B*, and *W* are of appropriate dimensions. The output *y* is interpreted as muscle activity.

All datasets were from the original simulations analyzed in those publications, with the exception of the RNN model of [36]. We re-simulated that model based on similar procedures described in [36]. After stabilizing the network using their procedure, we needed to specify each of the 72 initial states *x*(1,*c*) (one for each condition). We first computed the controllability Gramian of the linearized network (the matrix *Q* in [36]). The orthonormal columns of *Q* correspond to potential choices of initial states; the first column is an initial state that evokes the ‘strongest’ response (in terms of the total energy of the corresponding signals *r*); the second column gives the next strongest, and so forth. We selected the initial state for each condition to roughly match the temporal pattern of total energy (summed across all neurons) of the empirical neural data. Namely, we first considered the instantaneous power *P*(*t*) ≔ *r*(*t*)^⊤^*r*(*t*). Next, for a given column of *Q* (a possible choice of initial state), we simulated the network and measured the correlation across times between *P*(*t*) of the simulated data and *P*(*t*) of the empirical data for a given condition. After determining the 5 columns of *Q* that yielded the highest correlations, we chose each *x*(1,*c*) to be the weighted sum of those 5 columns that best matched *P*(*t*) for that condition. The net effect of this procedure was to produce a rich set of dynamics, flowing from 72 initial states, that provided a possible basis set for producing patterns of EMG for the 72 conditions. We confirmed the network did indeed provide such a basis set (*e*.*g*., that the EMG could be fit as a weighted sum of the responses in the network).

### Data preprocessing

For all experimental neural data, spike trains were smoothed with a Gaussian kernel (20 ms standard deviation) and sampled every 10 ms. Firing rate values were averaged across trials resulting in a population tensor of size *N* × *C* × *T*. Each element of this tensor is simply the firing rate for the corresponding neuron, condition and time. To ensure that analysis was not dominated by a few high-rate neurons, we normalized firing rates. Because normalization can occasionally lead to an undesirable expansion of sampling noise for low-rate neurons, we employed a ‘soft-normalization’ procedure (this same normalization is used in [30]). Each neuron was normalized according to:

$$x_{n}(c,t)\leftarrow \frac{x_{n}(c,t)}{5+{\text{range}}_{c,t}(x_{n}(c,t))},$$ (8)

where *i* = 1,…,*N*. The function range_*c*,*t*_(⋅) returns the difference between the maximum and minimum firing rates across all conditions and times for a given neuron. The soft normalization constant 5 mapped high firing rate neurons (*e*.*g*., 100 Hz) to a new range close to one. Low firing rate neurons were mapped to a range somewhat less than one (*e*.*g*., a neuron with a range of 5 spikes/s would be mapped to a new range of 0.5). This preprocessing allows neurons to contribute roughly equally regardless of their firing rate range. This is especially desirable when analyses involve the mean squared error. For example, without normalization the same relative error will be 25 times greater for a neuron with a 0–100 Hz firing rate range relative to a neuron with a 0–20 Hz firing rate range. That said, we emphasize that our results (*e*.*g*., the preferred mode of a given dataset) did not depend on the choice of soft normalization constant.

We wished to analyze temporal response structure that was different across conditions. We therefore removed the ‘cross-condition mean’ from the entire population tensor. We averaged the tensor across conditions resulting in an *N* × *T* matrix that we subtracted from every *N* × *T* matrix of data. This is related to the standard PCA step of first removing the mean value of each variable, and ensured that the analysis did not consider response structure that was identical across conditions, such as an elevation of firing rates for all visual stimuli or all reach directions.

All datasets naturally had an unequal number of neurons (*N*) and conditions (*C*). To ensure that basis-neuron and basis-condition reconstructions were compared on similar footing, we removed excess neurons or conditions in each dataset so that *N* = *C*. In most datasets there were more neurons than conditions. In such cases we kept the *N* = *C* neurons with the highest ratio of signal to noise. In the V1 dataset of **Fig 1A** there were more conditions than neurons. In this case we retained the *N* = *C* conditions that elicited the most temporal complexity in the population response (assessed via the standard deviation of the firing rate across all neurons and times). The specific preprocessing choices (filter length, normalization, equalizing *N* and *C*) were made to minimize any potential bias toward basis-neurons or basis-conditions. Still, none of these choices were found the affect the outcome of the analyses.

### Preferred-mode analysis

For each population tensor $X\in {\mathbb{R}}^{N\times C\times T}$ we quantified how well it could be reconstructed from a small set of *k* basis-neurons or *k* basis-conditions (the method for choosing *k* is described later). To illustrate, we first consider the case of basis-neurons (the case of basis-conditions is entirely parallel). Each of the recorded neurons is a set of *T* datapoints (one per time) for *C* conditions and thus forms a *C* × *T* matrix. Each basis neuron is also a *C* × *T* matrix. The data for each of the *N* neurons (each *C* × *T* matrix within the full population tensor) was approximated as a weighted sum of *k* basis-neuron matrices. Weights and basis neurons were chosen to provide the reconstruction with the lowest error.

To find those weights and basis neurons we applied SVD along the neuron mode of the population tensor. This procedure amounts to ‘unfolding’ (or reshaping) the tensor into a matrix, ***X***_(1)_ ∈ ℝ^*N*×*CT*^, where the subscript in parentheses indicates which mode appears as the row index in the matrix (see [49]). The order in which the columns appear in the matrix does not affect our analysis. We applied the SVD to ***X***_(1)_. The right singular vectors of ***X***_(1)_ correspond to vectors of dimension *CT*, which can be reshaped into *C* × *T* matrices corresponding to ‘basis-neurons.’ The singular values (squared) of ***X***_(1)_ indicate how much variance is explained by each basis-neuron. The approach to finding basis-conditions is parallel to the above and involves the SVD of ***X***_(2)_ ∈ ℝ^*C*×*NT*^. For both reconstructions we assessed the mean squared error between the elements of the original tensor and those of the reconstructed tensor. The reconstructed tensor was produced by multiplying the matrices produced by the SVD after appropriately limiting the inner dimensions based on the number of basis elements *k*. For example, if ***X***_(1)_ = *USV*^⊤^, then $X_{\left(1\right)}^{\text{rec}}=U_{:,1:k}S_{1:k,1:k}V_{1:k,:}^{⊤}$. We note that for practical convenience reconstruction error can also be readily computed from the first *k* singular values. For visualization we express reconstruction error in normalized form, relative to the total variance of the data.

We extended the above analysis to quantify reconstruction error as a function of the number of time-points included in the tensor (**Figs 3,4 and 6**). We began by considering a single time-point halfway through the response: *t*_half_ = round(*T*/2). We used this time to ask how many basis elements (basis-neurons and basis-conditions) were necessary to achieve low reconstruction error. As above we applied the SVD, in this case to the matrix $X_{:,:,t_{\text{half}}}\in {\mathbb{R}}^{N\times C\times 1}$. We chose the smallest number *k* such that normalized reconstruction error using the first *k* basis elements was less than 5%. Because $X_{:,:,t_{\text{half}}}$ is a matrix, the value of *k* is the same for basis-neurons and basis-conditions. We then analyzed $X_{:,:,t_{\text{half}}-1:t_{\text{half}}+1}\in {\mathbb{R}}^{N\times C\times 3}$ and quantified reconstruction error when using *k* basis-neurons versus *k* basis-conditions (*i*.*e*., the standard procedure described above was applied, but to a tensor that contained three times rather than all times). We repeated this for $X_{:,:,t_{\text{half}}-2:t_{\text{half}}+2}\in {\mathbb{R}}^{N\times C\times 5}$ and so forth until the full *N* × *C* × *T* tensor was analyzed.

To assess statistical reliability, we computed reconstruction error independently for each condition. This yielded a distribution of errors with a given mean and standard error. It is that mean and standard error that are plotted in **Figs 2C**, **3C, 3E**, **4 and 6**, and the right columns of **Fig 8**. We chose to compute the standard error across conditions rather than across both neurons and conditions to be conservative (the latter would have yielded even smaller error bars).

### Control datasets and analyses

We performed a three control analyses to assess the robustness of the central method. The outcome of the first of these is shown in the Results; the outcome of the other two are shown here. First, we analyzed two control datasets intentionally constructed to have surface-level features similar to the original empirical datasets. To generate the manipulated V1 dataset, we first extracted the top 24 basis-conditions (out of 25) from the original dataset using SVD. We randomly partitioned the basis set into 6 partitions (4 elements each), and summed the elements within a partition to create a single basis-condition, resulting in 6 total basis-conditions. We then reconstructed the manipulated dataset neuron-by-neuron: each new neuron was a least-squares fit to the original neuron, but using the 6 basis-conditions derived above. This ensured that the manipulated V1 data had relatively few degrees of freedom across conditions, yet resembled the original V1 neurons in terms of basic response properties. The manipulated M1 dataset was constructed analogously, but using 6 basis-neurons derived from the original 72. The outcome of this analysis is shown in **Fig 5**.

**Fig 9:**
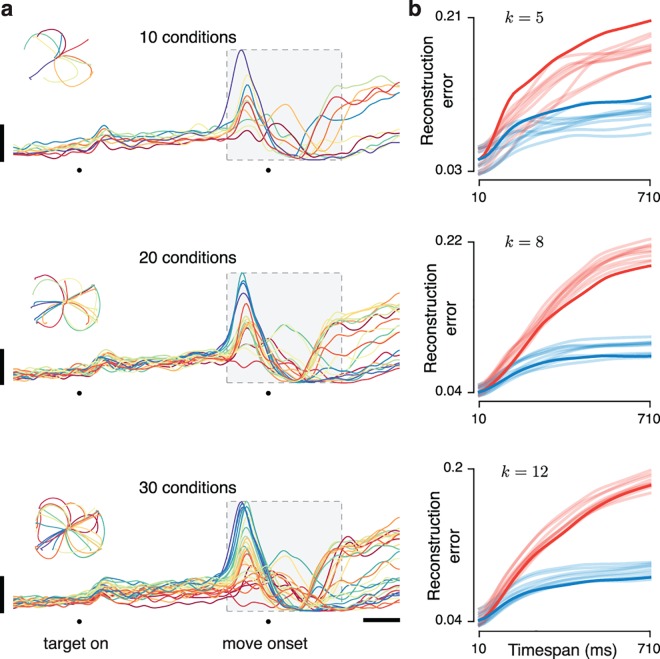
Preferred-mode analysis using a variable number of conditions. (**a**) Responses of one example neuron illustrating an instance of randomly selected sets of 10 (top), 20 (middle), and 30 (bottom) conditions. Horizontal and vertical calibration bars correspond to 200 ms and 20 spikes/s. (**b**) Reconstruction error as a function of timespan for sets of 10 (top), 20 (middle), and 30 (bottom) conditions. Multiple traces are shown: one each for 10 draws of random conditions. Dark traces show the neuron-mode (red) and condition-mode (blue) reconstruction error for the particular sets of conditions illustrated in **a**. Even for small numbers of conditions (as few as 10) there was a consistent preferred mode. In fact, the preferred mode was even more consistent than it appears, as the comparisons are naturally paired: every red trace has a corresponding blue trace. These tended to move upwards and downwards together (as in the example illustrated with the dark traces) with a reasonably consistent difference between them.

Second, to assess robustness of the central method with respect to the number of recorded conditions, we repeated the analysis for one M1 dataset (the dataset from **Fig 3E**) that originally contained 72 conditions. We down-sampled the data by selecting 10, 20, and 30 conditions. Conditions were selected randomly, but via a procedure that also ensured that the selected conditions were sufficiently different (*e*.*g*., that they were not all rightwards reaches). The preferred mode was indeed robust even when the number of conditions was reduced (**Fig 9**).

**Fig 10:**
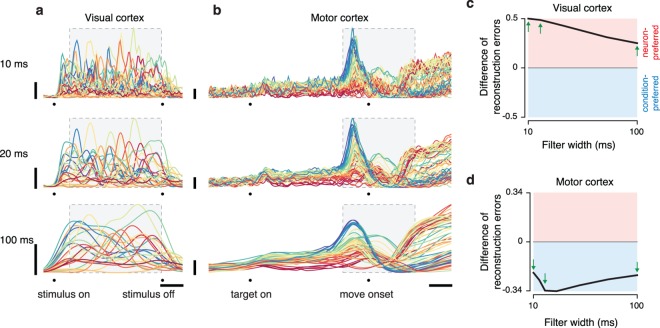
Effect of spike filtering width on the preferred mode. Spike trains from V1 and M1 datasets were filtered with a Gaussian kernel of varying widths (width corresponds to the standard deviation of the Gaussian). (**a**) Response of one example V1 neuron for filter widths of 10 ms, 20 ms (the default value used for all other analyses in this study), and 100 ms. (**b**) Response of one example M1 neuron for the same three filter widths. Horizontal and vertical calibration bars correspond to 200 ms and 20 spikes/s. (**c**) Difference in reconstruction error between the condition mode and the neuron mode (computed as in **Fig 7**) as a function of filter width, for the V1 dataset from panel **a**. Differences are positive, indicating that the neuron mode incurred less error and is preferred. Green arrows indicate filter widths of 10, 20, and 100, corresponding to the examples shown in **a**. (**d**) Difference in reconstruction error for the M1 dataset from panel **b**. Differences are negative, indicating that the condition mode incurred less error and is preferred. Thus, the preferred mode is robust to filter width, despite the wide range of frequencies highlighted or suppressed by filter width choices.

Finally, we analyzed the effect of spike filter widths on the preferred mode for the V1 and M1 datasets (**Fig 10**). This analysis served two purposes. First, spike filtering is a standard preprocessing step and we wanted to ensure that results were not dependent on the particular choice of filter width. Second, the analysis reveals that the preferred mode is not in some way to due to the smoothness or frequency content of neural signals—a potential concern when comparing brain areas whose neurons have fundamentally different response properties, as is the case with V1 and M1.

### Linear Models

In **Fig 8**we illustrated some basic properties of the preferred mode using simulations of linear dynamical systems (Eq (6)). These simple simulations were separate from the simulations of published models described above. For these simple simulations we chose *N* = *C* = 20, and *T* = 300. We set *M* = 10 (*i*.*e*. the input *u* was ten-dimensional). We first generated the matrices *A* and *B* with orthonormal columns; for *A*, eigenvalues were random but were clustered near 1 to ensure smooth trajectories for our choice of *T* (this was not a necessary step, but yielded roughly comparable oscillation frequencies to those observed in the datasets of **Fig 4**). Each input *u*_*m*_ was composed of a randomly weighted sum of 20 sinusoids. Sinusoid frequency was determined by the same procedure that generated the eigenvalues of *A*. Thus, inputs had the same frequency components as the dynamics, ensuring similar single-neuron response properties across simulations. Initial states across conditions were chosen randomly and were constrained to span 10 dimensions. With these parameters fixed, we simulated the system *x*(*t* + 1,*c*) = *aAx*(*t*,*c*) + *bBu*(*t*,*c*), where *a* ∈ [0,1] and *b* ∈ [0,1] determined the strength of dynamics and inputs, respectively. In **Fig 8A–8D**, values of *a* were 0, 0.98, 0.99, and 1 (Note that values of *a* even slightly lower than unity lead to rapidly decaying ‘weak’ dynamics). Values of *b* were 1, 0.05, 0.03, and 0 (note that inputs need to be quite weak before they cease to have a strong effect on a system with persistent dynamics). Each panel in **Fig 8**involved the same choices of *A* and *B*, and the same initial states.

Data in **Fig 8E–8H** were simulated as above, with *a* = 1 and *b* = 0. However, the ‘data’ for which the preferred mode was computed consisted not of the values of the dynamic variable *x*, but rather of the values of an observation variable *y*. We treated *y* as the neural population being driven by ‘observing’ the dynamic state variable *x*, with *y*(*c*,*t*) = *Cx*(*c*,*t*). The observation matrix *C* had different ranks depending on how fully *y* reflected *x*. Specifically, *C* was diagonal with 1s on the first 3, 4, 8, and 20 diagonal entries for **Fig 8**panels **e,f,g,h**, respectively (and 0s elsewhere).

### Derivation of the preferred mode for idealized models

Here we show that neuron-preferred structure is expected when responses are driven by unconstrained external variables, while condition-preferred structure is expected when neural responses are shaped by internal dynamics. We consider a dataset $X\in {\mathbb{R}}^{N\times C\times T}$, where *N*, *C* and *T* are the number of recorded neurons, experimental conditions, and times. We also consider a set of external signals, or inputs, $U\in {\mathbb{R}}^{M\times C\times T}$, where *M* is the number of external variables. The column vector *x*(*t*,*c*) ∈ ℝ^*N*^ is the firing rate of every neuron at time *t* ∈ {1,…,*T*} for condition *c* ∈ {1,…,*C*}. An *N* × *C* matrix ‘slice’ of X is denoted *X*(*t*) ∈ ℝ^*N*×*C*^, and is the population state across all conditions for time *t*. We define the ‘mode-1’ and ‘mode-2’ matrix unfoldings of X:

$$X_{\left(1\right)}≔[\begin{array}{cccc} X(1) & X(2) & \cdots & X(T) \end{array}]\in {\mathbb{R}}^{N\times CT},$$ (9)

$$X_{\left(2\right)}≔[\begin{array}{cccc} X{\left(1\right)}^{⊤} & X{\left(2\right)}^{⊤} & \cdots & X{\left(T\right)}^{⊤} \end{array}]\in {\mathbb{R}}^{C\times NT}.$$

Each row of ***X***_(1)_ corresponds to one neuron, and each row of ***X***_(2)_ corresponds to one condition. Importantly, rank(***X***_(1)_) is the number of basis-neurons needed to reconstruct X. Similarly, rank(***X***_(2)_) is the number of basis-conditions needed to reconstruct X.

**Definition**: A dataset $X\in {\mathbb{R}}^{N\times C\times T}$ is called neuron-preferred (condition-preferred) when the rank of the matrix unfolding ***X***_(1)_ (***X***_(2)_) of its sub-tensors $X_{T_{i}}\in {\mathbb{R}}^{N\times C\times T_{i}}$ does not increase with *T*_*i*_, while the rank of ***X***_(2)_ (***X***_(1)_) does increase with *T*_*i*_.

We evaluate the rank of each unfolding in datasets X generated by the following model classes:

x(t,c)=Bu(t,c), (10)

and

x(t+1,c)=Ax(t,c). (11)

We term Eq (10) the tuning model class (*B* ∈ ℝ^*N*×*M*^ defines each neuron’s tuning for external variables), and Eq (11) the dynamical model class (*A* ∈ ℝ^*N*×*N*^ specifies linear dynamics).

**Claim**: Models of the form Eq (10) (Eq (11)) generate datasets having neuron-preferred (condition-preferred) structure.

### Part 1: The tuning model class implies neuron-preferred structure

To begin, note that Eq(10) can be written as a matrix equation,

X(t)=BU(t). (12)

For any *T*_*i*_ ∈ {1,…,*T*}, Eq (12) implies,

$$[\begin{array}{cccc} X(1) & X(2) & \cdots & X(T_{i}) \end{array}]=B[\begin{array}{cccc} U(1) & U(2) & \cdots & U(T_{i}) \end{array}],$$ (13)

or, more compactly, ***X***_(1)_ = *B****U***_(1)_. For the mode-2 unfolding, given Eq (12) we can also write,

$$\left[\begin{array}{c} X(1)\\ X(2)\\ \vdots \\ X(T_{i}) \end{array}\right]=\left[\begin{array}{cccc} B & 0 & \cdots & 0\\ 0 & B & \cdots & 0\\ \vdots & \vdots & \ddots & \vdots \\ 0 & 0 & \cdots & B \end{array}\right]\left[\begin{array}{c} U(1)\\ U(2)\\ \vdots \\ U(T_{i}) \end{array}\right],$$ (14)

*i*.*e*., $X_{\left(2\right)}=U_{\left(2\right)}(I_{T_{i}}⊗B^{⊤})$ where $I_{T_{i}}$ is the *T*_*i*_ × *T*_*i*_ identity matrix and ⊗ denotes the Kronecker product. Thus,

$$x(t,c)=Bu(t,c)⟺X_{\left(1\right)}=BU_{\left(1\right)}⟺X_{\left(2\right)}=U_{\left(2\right)}(I_{T_{i}}⊗B^{⊤}).$$ (15)

We can take without loss of generality rank(*B*) = *M*. Thus, rank(***X***_(1)_) = rank(*B****U***_(1)_) = min(*M*,rank(***U***_(1)_)) ≤ *M*. On the other hand rank(***X***_(2)_) = rank(***U***_(2)_) ≤ min(*C*,*MT*_*i*_). (To see this note that ***U***_(2)_ is size *C* × *MT*_*i*_ and $\left(I_{T_{i}}⊗B^{⊤}\right)$ is size *MT*_*i*_ × *NT*_*i*_ and full rank). Thus, the rank of the mode-1 unfolding is strictly bounded by *M* (which is fixed by the model) while the rank of the mode-2 unfolding can grow arbitrarily with *C* and *T*_*i*_ (which can be increased by the experimenter). Thus, datasets generated by the tuning model class are neuron-preferred when the inputs are unconstrained, *i*.*e*. when rank(***U***_(2)_) grows beyond *M* with increasing *T*_*i*_. This shows part 1 of the claim.

### Part 2: The dynamical model class implies condition-preferred structure

Eq (11) can be written *X*(*t* + 1) = *AX*(*t*), which admits the solution

$$X(t)=A^{t-1}X(1),$$ (16)

where the matrix *A*^*t*−1^ maps initial states to the state at time *t*. We define the tensor $A\in {\mathbb{R}}^{N\times N\times T}$ to be the collection of all matrices *A*^*t*−1^ for *t* = 1,…,*T* (from here, the definitions of ***A***_(1)_ and ***A***_(2)_ follow). We can now write

$$\left[\begin{array}{c} X(1)\\ X(2)\\ \vdots \\ X(T_{i}) \end{array}\right]=\left[\begin{array}{c} I_{N}\\ A\\ \vdots \\ A^{T_{i}-1} \end{array}\right]X(1).$$ (17)

More compactly: ***X***_(2)_ = *X*(1)^⊤^***A***_(2)_. To find ***X***_(1)_, given Eq (16) we can write

$$[\begin{array}{cccc} X(1) & X(2) & \cdots & X(T_{i}) \end{array}]=[\begin{array}{cccc} I_{N} & A & \cdots & A^{T_{i}-1} \end{array}]\left[\begin{array}{cccc} X(1) & 0 & \cdots & 0\\ 0 & X(1) & \cdots & 0\\ \vdots & \vdots & \ddots & \vdots \\ 0 & 0 & \cdots & X(1) \end{array}\right].$$ (18)

More compactly: $X_{\left(1\right)}=A_{\left(1\right)}(I_{T_{i}}⊗X(1))$. Thus,

$$x(t+1,c)=Ax(t,c)⟺X_{\left(1\right)}=A_{\left(1\right)}(I_{T_{i}}⊗X(1))⟺X_{\left(2\right)}=X{\left(1\right)}^{⊤}A_{\left(2\right)}.$$ (19)

We note that the rank of the mode-1 unfolding can grow with *T*_*i*_,

$$rank(X(1))\leq rank(\left[X(1)\;AX(1)\right])\leq rank(\left[X(1)\;AX(1)\;A^{2}X(1)\right])\leq \cdots ,$$ (20)

and can eventually reach the maximum of rank(*A*) (due to the Cayley-Hamilton theorem). On the other hand, rank(***X***_(2)_) = rank(*X*(1)), where equality follows because *X*(1)^⊤^ is a submatrix of ***X***_(2)_. The rank of the mode-2 unfolding thus does not grow with *T*_*i*_. Therefore, datasets generated by the dynamical model class are condition-preferred when rank([*X*(1) *AX*(1)]) > rank(*X*(1)), *i*.*e*. whenever the matrix *A* maps the initial states into a subspace not spanned by the columns of *X*(1). This completes part 2 of the claim.

### Low-rank assumptions pertaining to the above derivation

Given the above, a natural expectation is that *X*(*t*) = *BU*(*t*) ⇒ rank(***X***_(1)_) ≤ rank(***X***_(2)_) with rank(***X***_(2)_) growing as more times are considered. Similarly one expects *X*(*t* + 1) = *AX*(*t*) ⇒ rank(***X***_(2)_) ≤ rank(***X***_(1)_) with rank(***X***_(1)_) growing as more times are considered. These expectations will indeed hold given reasonable low-rank assumptions. The first inference (that tuning models imply a neuron-mode preference) depends upon recording more neurons and conditions than the presumed number of represented variables, *i*.*e*., we need *N* > *M* and *C* > *M*. Otherwise it is possible for min(*C*,*MT*_*i*_) (the limit on rank(***X***_(2)_)) to be smaller than *M* (the limit on rank(***X***_(1)_)). In practice, the adequacy of the data can be evaluated by testing whether results change when more neurons/conditions are added. Importantly, the present results did not depend upon neuron/condition count. For example, effects are equally strong in **Fig 4F** and **Fig 4G** despite a threefold difference in the number of analyzed neurons and conditions. Still, the possibility of data being neuron- or condition-limited is a real one, and provides strong motivation to analyze datasets with many neurons and many diverse conditions.

The second inference (dynamical models imply a condition-mode preference) depends upon the assumption rank(*X*(1)) < rank(*A*). In other words, the set of initial states (one per condition) must occupy a proper subspace of all states visited as the dynamics governed by *A* unfold. Otherwise rank(***X***_(1)_) = rank(***X***_(2)_) regardless of how many times are considered (*i*.*e*., the red and blue traces in **Fig 4**would be equal and would not rise with time). In practice the assumption rank(*X*(1)) < rank(*A*) is reasonable, both because we never observed the above signature for any dataset and because we have recently shown that M1/PMd preparatory states do not occupy all dimensions subsequently explored during movement [61].

In summary, the key low-rank assumptions are likely to be valid when considering many neurons and diverse conditions. Models of the form *X*(*t*) = *BU*(*t*) will thus have a stable rank(***X***_(1)_) and an unstable rank(***X***_(2)_). Models of the form *X*(*t* + 1) = *AX*(*t*) will have a stable rank(***X***_(2)_) and an unstable rank(***X***_(1)_). The converse inferences will also hold. If rank(***X***_(1)_) is stable as times are added then the data can be factored as in Eq (13) and thus modeled as *X*(*t*) = *BU*(*t*). If rank(***X***_(2)_) is stable then the data can be factored as in Eq (17) (possibly requiring a time-varying *A*) and thus modeled as *X*(*t* + 1) = *AX*(*t*).

### Time-varying dynamics

Part 2 of the above claim extends naturally to the equation *X*(*t* + 1) = *A*(*t*)*X*(*t*), a time-varying linear dynamical system. As long as the dynamics—the (potentially time-varying) vector fields—are the same across conditions then the above arguments hold. Thus, while the appearance of condition-preferred structure depends on the constraints imposed by dynamics, such structure does not depend on time-invariant dynamics. Because dynamical systems can often be approximated as time-varying linear systems (especially over short timescales), condition-preferred structure is likely to be common whenever population structure is shaped by strong dynamics.

### Measuring rank

Empirical neural data inevitably include sampling noise in the estimated firing rates, due to finite trial-counts from spiking neurons. Similarly, some degree of nonlinearity is always present in the form of spiking thresholds or deeper nonlinearities in the underling representations or dynamics. Thus, the measured ***X***_(1)_ and ***X***_(2)_ will always be full rank. In practice, we therefore evaluated not the ranks of ***X***_(1)_ and ***X***_(2)_ *per se* but the success of rank-*k* reconstructions of ***X***_(1)_ and ***X***_(2)_. In simulations we found that this approach works well. Reconstruction error is increased by the addition of noise or nonlinearities, but this occurs approximately equally for both ***X***_(1)_ and ***X***_(2)_. Thus, the preferred-mode analysis is still able to successfully differentiate datasets generated by static nonlinear tuning models from autonomous nonlinear dynamical models (*e*.*g*., **Fig 4**).

## References

[1] Churchland MM, Shenoy KV. Temporal complexity and heterogeneity of single-neuron activity in premotor and motor cortex. J Neurophysiol. 2007;97: 4235–4257. 10.1152/jn.00095.2007 17376854

[2] Raposo D, Kaufman MT, Churchland AK. A category-free neural population supports evolving demands during decision-making. Nat Neurosci. Nature Publishing Group; 2014;17: 1784–1792. 10.1038/nn.3865 25383902 PMC4294797

[3] Bair W, Koch C. Temporal precision of spike trains in extrastriate cortex of the behaving macaque monkey. Neural Comput. 1996. 10.1162/neco.1996.8.6.1185 8768391

[4] Benucci A, Ringach DL, Carandini M. Coding of stimulus sequences by population responses in visual cortex. Nat Neurosci. 2009;12: 1317–1324. 10.1038/nn.2398 19749748 PMC2847499

[5] Grillner S. Biological pattern generation: the cellular and computational logic of networks in motion. Neuron. 2006;52: 751–766. 10.1016/j.neuron.2006.11.008 17145498

[6] Priebe NJ, Lisberger SG. Constraints on the source of short-term motion adaptation in macaque area MT. II. tuning of neural circuit mechanisms. J Neurophysiol. 2002;88: 370–382. 12091561 10.1152/jn.2002.88.1.370 PMC2581620

[7] Tong L, Liu RW, Soon VC, Huang YF. Indeterminacy and identifiability of blind identification. IEEE Trans Circuits Syst. 1991;38: 499–509.

[8] Wu W, Hatsopoulos N. Evidence against a single coordinate system representation in the motor cortex. Exp Brain Res. Springer-Verlag; 2006;175: 197–210. 10.1007/s00221-006-0556-x 16775704

[9] Hatsopoulos NG, Xu Q, Amit Y. Encoding of movement fragments in the motor cortex. J Neurosci. 2007;27: 5105–5114. 10.1523/JNEUROSCI.3570-06.2007 17494696 PMC6672361

[10] Fu QG, Flament D, Coltz JD, Ebner TJ. Temporal encoding of movement kinematics in the discharge of primate primary motor and premotor neurons. J Neurophysiol. 1995;73: 836–854. 7760138 10.1152/jn.1995.73.2.836

[11] Mussa-Ivaldi FA. Do neurons in the motor cortex encode movement direction? An alternative hypothesis. Neurosci Lett. 1988;91: 106–111. 3173781 10.1016/0304-3940(88)90257-1

[12] Fetz EE. Are movement parameters recognizably coded in the activity of single neurons. Behavioral and Brain Sciences. 1992;15: 679–690.

[13] Sanger TD. Theoretical Considerations for the Analysis of Population Coding in Motor Cortex. Neural Comput. 1994;6: 29–37.

[14] Todorov E. Direct cortical control of muscle activation in voluntary arm movements: a model. Nat Neurosci. 2000;3: 391–398. 10.1038/73964 10725930

[15] Reimer J, Hatsopoulos NG. The problem of parametric neural coding in the motor system. Adv Exp Med Biol. Boston, MA: Springer US; 2009;629: 243–259. 10.1007/978-0-387-77064-2_12 19227503 PMC4480635

[16] Scott SH. Inconvenient truths about neural processing in primary motor cortex. J Physiol. 2008;586: 1217–1224. 10.1113/jphysiol.2007.146068 18187462 PMC2375659

[17] Scott SH. Population vectors and motor cortex: neural coding or epiphenomenon? Nature Publishing Group. 2000;3: 307–308. 10.1038/73859 10725914

[18] Graziano MSA, Aflalo TN. Mapping behavioral repertoire onto the cortex. Neuron. 2007;56: 239–251. 10.1016/j.neuron.2007.09.013 17964243

[19] Georgopoulos AP, Carpenter AF. Coding of movements in the motor cortex. Curr Opin Neurobiol. 2015;33C: 34–39. 10.1016/j.conb.2015.01.012 25646932

[20] Chase SM, Schwartz AB. Inference from populations: going beyond models. Prog Brain Res. 2011;192: 103–112. 10.1016/B978-0-444-53355-5.00007-5 21763521

[21] Kalaska JF. From intention to action: motor cortex and the control of reaching movements. Adv Exp Med Biol. 2009;629: 139–178. 10.1007/978-0-387-77064-2_8 19227499

[22] Scott SH, Kalaska JF. Reaching movements with similar hand paths but different arm orientations. I. Activity of individual cells in motor cortex. J Neurophysiol. 1997;77: 826–852. 9065853 10.1152/jn.1997.77.2.826

[23] Kakei S, Hoffman DS, Strick PL. Muscle and movement representations in the primary motor cortex. Science. 1999;285: 2136–2139. 10497133 10.1126/science.285.5436.2136

[24] Caminiti R, Johnson PB, Burnod Y, Galli C. Shift of preferred directions of premotor cortical cells with arm movements performed across the workspace. Experimental brain …. 1990. 10.1007/BF00232214 2073945

[25] Ashe J, Georgopoulos AP. Movement parameters and neural activity in motor cortex and area 5. Cereb Cortex. 1994;4: 590–600. 7703686 10.1093/cercor/4.6.590

[26] Ajemian R, Bullock D, Grossberg S. Kinematic coordinates in which motor cortical cells encode movement direction. J Neurophysiol. 2000;84: 2191–2203. 11067965 10.1152/jn.2000.84.5.2191

[27] Ajemian R, Green A, Bullock D, Sergio L, Kalaska J, Grossberg S. Assessing the Function of Motor Cortex: Single-Neuron Models of How Neural Response Is Modulated by Limb Biomechanics. Neuron. 2008;58: 414–428. 10.1016/j.neuron.2008.02.033 18466751

[28] Sergio LE, Hamel-Pâquet C, Kalaska JF. Motor cortex neural correlates of output kinematics and kinetics during isometric-force and arm-reaching tasks. J Neurophysiol. 2005;94: 2353–2378. 10.1152/jn.00989.2004 15888522

[29] Churchland MM, Cunningham JP, Kaufman MT, Ryu SI, Shenoy KV. Cortical preparatory activity: representation of movement or first cog in a dynamical machine? Neuron. 2010;68: 387–400. 10.1016/j.neuron.2010.09.015 21040842 PMC2991102

[30] Churchland MM, Cunningham JP, Kaufman MT, Foster JD, Nuyujukian P, Ryu SI, et al Neural population dynamics during reaching. Nature. 2012;487: 51–56. 10.1038/nature11129 22722855 PMC3393826

[31] Shenoy KV, Sahani M, Churchland MM. Cortical control of arm movements: a dynamical systems perspective. Annu Rev Neurosci. 2013;36: 337–359. 10.1146/annurev-neuro-062111-150509 23725001

[32] Churchland MM, Cunningham JP. A Dynamical Basis Set for Generating Reaches. Cold Spring Harb Symp Quant Biol. 2014;79: 67–80. 10.1101/sqb.2014.79.024703 25851506

[33] Vaidya M, Kording K, Saleh M, Takahashi K, Hatsopoulos NG. Neural coordination during reach-to-grasp. J Neurophysiol. American Physiological Society; 2015;114: 1827–1836. 10.1152/jn.00349.2015 26224773 PMC4575972

[34] Sussillo D, Churchland MM, Kaufman MT, Shenoy KV. A neural network that finds a naturalistic solution for the production of muscle activity. Nat Neurosci. 2015;18: 1025–1033. 10.1038/nn.4042 26075643 PMC5113297

[35] Maier MA, Shupe LE, Fetz EE. Dynamic Neural Network Models of the Premotoneuronal Circuitry Controlling Wrist Movements in Primates. J Comput Neurosci. Kluwer Academic Publishers; 2005;19: 125–146. 10.1007/s10827-005-0899-5 16133816

[36] Hennequin G, Vogels TP, Gerstner W. Optimal control of transient dynamics in balanced networks supports generation of complex movements. Neuron. 2014;82: 1394–1406. 10.1016/j.neuron.2014.04.045 24945778 PMC6364799

[37] Lillicrap TP, Scott SH. Preference distributions of primary motor cortex neurons reflect control solutions optimized for limb biomechanics. Neuron. 2013;77: 168–179. 10.1016/j.neuron.2012.10.041 23312524

[38] Scott SH. Optimal feedback control and the neural basis of volitional motor control. Nat Rev Neurosci. 2004;5: 532–546. 15208695 10.1038/nrn1427

[39] Todorov E, Jordan MI. Optimal feedback control as a theory of motor coordination. Nature Publishing Group. 2002;5: 1226–1235. 10.1038/nn963 12404008

[40] Schieber MH, Rivlis G. Partial reconstruction of muscle activity from a pruned network of diverse motor cortex neurons. J Neurophysiol. 2007;97: 70–82. 10.1152/jn.00544.2006 17035361

[41] Pohlmeyer EA, Solla SA, Perreault EJ, Miller LE. Prediction of upper limb muscle activity from motor cortical discharge during reaching. Journal of Neural Engineering. 2007;4: 369–379. 10.1088/1741-2560/4/4/003 18057504 PMC2586074

[42] Hatsopoulos NG. Encoding in the motor cortex: was evarts right after all? Focus on "motor cortex neural correlates of output kinematics and kinetics during isometric-force and arm-reaching tasks". J Neurophysiol. 2005;94: 2261–2262. 10.1152/jn.00533.2005 16160087

[43] Morrow MM, Pohlmeyer EA, Miller LE. Control of muscle synergies by cortical ensembles. Adv Exp Med Biol. Boston, MA: Springer US; 2009;629: 179–199. 10.1007/978-0-387-77064-2_9 19227500

[44] Georgopoulos AP, Naselaris T, Merchant H, Amirikian B. Reply to Kurtzer and Herter. J Neurophysiol. American Physiological Society; 2007;97: 4391–4392.

[45] Moran DW, Schwartz AB. One motor cortex, two different views. Nature Publishing Group. 2000;3: 963–author reply 963–5. 10.1038/79880 11017157

[46] Pearce TM, Moran DW. Strategy-dependent encoding of planned arm movements in the dorsal premotor cortex. Science. 2012;337: 984–988. 10.1126/science.1220642 22821987 PMC3667666

[47] Cunningham JP, Yu BM. Dimensionality reduction for large-scale neural recordings. Nat Neurosci. Nature Publishing Group; 2014;17: 1500–1509. 10.1038/nn.3776 25151264 PMC4433019

[48] Sadtler PT, Quick KM, Golub MD, Chase SM, Ryu SI, Tyler-Kabara EC, et al Neural constraints on learning. Nature. Nature Publishing Group; 2014;512: 423–426. 10.1038/nature13665 25164754 PMC4393644

[49] Kolda TG, Bader BW. Tensor Decompositions and Applications. SIAM Rev. 2009;51: 455.

[50] Benucci A, Saleem AB, Carandini M. Adaptation maintains population homeostasis in primary visual cortex. Nat Neurosci. Nature Publishing Group; 2013;16: 724–729. 10.1038/nn.3382 23603708 PMC3665725

[51] Moran DW, Schwartz AB. Motor cortical representation of speed and direction during reaching. J Neurophysiol. 1999;82: 2676–2692. 10561437 10.1152/jn.1999.82.5.2676

[52] Georgopoulos AP, Ashe J. One motor cortex, two different views. Nature Publishing Group. 2000;3: 963–author reply 964–5. 10.1038/79882 11017158

[53] Hubel DH, Wiesel TN. Receptive fields of single neurones in the cat's striate cortex. J Physiol. 1959;587: 2721–2732. 10.1113/jphysiol.1959.sp006308 PMC1363130 14403679

[54] Rokni U, Sompolinsky H. How the brain generates movement. Neural Comput. MIT Press 55 Hayward Street, Cambridge, MA 02142–1315 email: journals-info@mit.edu; 2012;24: 289–331. 10.1162/NECO_a_00223 22023199

[55] Tanaka H, Sejnowski TJ. Computing reaching dynamics in motor cortex with Cartesian spatial coordinates. J Neurophysiol. 2013;109: 1182–1201. 10.1152/jn.00279.2012 23114209 PMC3569131

[56] Pruszynski JA, Scott SH. Optimal feedback control and the long-latency stretch response. Exp Brain Res. 2012;218: 341–359. -8 10.1007/s00221-012-3041-8 22370742

[57] Suminski AJ, Tkach DC, Fagg AH, Hatsopoulos NG. Incorporating feedback from multiple sensory modalities enhances brain-machine interface control. J Neurosci. Society for Neuroscience; 2010;30: 16777–16787. 10.1523/JNEUROSCI.3967-10.2010 21159949 PMC3046069

[58] London BM, Miller LE. Responses of somatosensory area 2 neurons to actively and passively generated limb movements. J Neurophysiol. 2013;109: 1505–1513. 10.1152/jn.00372.2012 23274308 PMC3774588

[59] Gold JI, Shadlen MN. The neural basis of decision making. Annu Rev Neurosci. 2007;30: 535–574. 10.1146/annurev.neuro.29.051605.113038 17600525

[60] Mante V, Sussillo D, Shenoy KV, Newsome WT. Context-dependent computation by recurrent dynamics in prefrontal cortex. Nature. 2013;503: 78–84. 10.1038/nature12742 24201281 PMC4121670

[61] Kaufman MT, Churchland MM, Ryu SI, Shenoy KV. Cortical activity in the null space: permitting preparation without movement. Nat Neurosci. 2014;17: 440–448. 10.1038/nn.3643 24487233 PMC3955357

